# A Three-Dimensional Framework for Rational Ion Separation with Polymeric Membrane Systems

**DOI:** 10.3390/membranes16090302

**Published:** 2026-09-15

**Authors:** George Bess-Palacios, Guillermo González-Sánchez, Claudia Alejandra Hernández-Escobar, Claudia Ivone Piñón-Balderrama, Miguel Alonso Orozco-Alvarado, América Susana Mares-García, Anayansi Estrada-Monje, Erasto Armando Zaragoza-Contreras

**Affiliations:** 1 Centro de Investigación en Materiales Avanzados, S.C., Miguel de Cervantes No. 120, Complejo Industrial Chihuahua, Chihuahua C.P. 31136, Mexico; george.bess@cimav.edu.mx (G.B.-P.); guillermo.gonzalez@cimav.edu.mx (G.G.-S.); claudia.hernandez@cimav.edu.mx (C.A.H.-E.); claudia.pinon@cimav.edu.mx (C.I.P.-B.); miguel.orozco@cimav.edu.mx (M.A.O.-A.); america.mares@cimav.edu.mx (A.S.M.-G.); 2 Centro de Innovación Aplicada en Tecnologías Competitivas, Omega No. 201, Industrial Delta, León C.P. 37545, Mexico; aestrada@ciatec.mx

**Keywords:** ion–ion selectivity, polymeric membranes, rational membrane design, structural plasticity, digital separation, physics-informed neural networks

## Abstract

This review treats selective ion separation in polymeric membrane systems as a design problem rather than as a fixed material property. To address the gap between laboratory performance and process-level relevance, a three-dimensional analytical framework that integrates transport mechanism, dynamic structural state, and separation-matrix complexity is proposed. Five research streams are compared: polymer inclusion membranes, ion-exchange membranes and electrodialysis, nanostructured and subnanometric frameworks, composite architectures, and polymer inclusion membrane – electrodialysis (PIM-ED) hybrids. The comparison shows that selectivity is governed not only by the nominal membrane mechanism, but also by hydration-driven structural changes and by the complexity of the operating matrix. Overall, the framework provides a common basis for interpreting selectivity, comparing membrane families, and guiding rational design for ion-separation processes.

## 1. Introduction

Selective separation of metal ions in aqueous solutions remains a major technical challenge in two dimensions. The demand for metals with defined electrochemical and functional properties has increased steadily over the last decade, driven by the energy transition, transport electrification, and the proliferation of energy storage devices [1,2]. Conversely, the contamination of water bodies by toxic metal ions, such as lead, cadmium, hexavalent chromium, and mercury, poses a global public health problem that conventional treatment processes resolve only partially [3,4]. In both contexts, the core technical challenge remains the same: the selective isolation of a specific ion from a complex aqueous mixture containing multiple cations with similar physicochemical properties.

Advances in high-performance desalination have relied in part on the incorporation of nanofillers into thin-film composite (TFC) architectures. Recent assessments of nanomaterial-modified membranes show that filler positioning, whether within the active layer, within the support, or at the interface, is a key determinant of permeability and fouling resistance. This evolution establishes the baseline from which ion selectivity must advance, moving beyond conventional water purification toward the precise discrimination of similar ionic species [5].

Relative to conventional ion separation methods, polymeric membranes can offer operational advantages, including continuous processing, a lower energy footprint, the absence of organic solvents in the process stream, and the potential for integration into compact, scalable systems [6]. However, their industrial adoption for high-selectivity ion separations remains limited. The limitation is not only technological but also conceptual. Specifically, the understanding of the mechanisms governing discrimination between ions of similar properties through polymeric matrices remains fragmentary, hindering the rational design of membranes and limiting performance prediction under real operating conditions [7,8]. This challenge has also motivated the systematic use of machine learning in membrane research [9].

Recent advances have often been addressed from partial perspectives. Some reviews have concentrated on specific membrane families; for example, polymer inclusion membranes (PIMs) utilizing ionic liquids as carriers have received systematic attention recently [10], and membranes with selectivity based on steric sieving and chemical affinity toward specific monovalent ions have been the subject of independent reviews [11,12]. Experimental work has demonstrated that modulating the hydrophobicity of pendant groups in polymeric micropores regulates pore hydration and, consequently, ion selectivity, establishing a direct link between molecular architecture and ionic discrimination [13]. A unified thermodynamic perspective is needed to connect these research streams. This review presents a comparative framework that contrasts electrostatic exclusion, steric-hydration sieving, and facilitated transport across diverse polymer families.

Rather than relying on single-variable evaluations, the analysis integrates the mechanistic foundations of nanoscale transport phenomena, recognizing that selective ion transport involves a complex convolution of Donnan exclusion, steric sieving, specific interactions, and hydration effects whose relative contributions vary under real operating conditions [7,14]. This baseline then serves to reconcile the theoretical tensions between models prioritizing pore-size control and those emphasizing specific chemical interactions as selectivity determinants. The analysis also addresses the macro-scale implications of structural plasticity, directly examining why the gap between laboratory-demonstrated selectivity and performance under real industrial conditions represents a scaling problem that current theory fails to integrate satisfactorily [15,16]. The potential of digital separation approaches is also considered, delineating how computational inverse design can bypass the empirical limitations of complex separation matrices.

The review is organized around three axes that progressively address these questions: fundamental transport and ion selectivity mechanisms (Section 3), the five research streams expressing these mechanisms in various membrane architectures (Section 4), and the critical integration of these streams into a conceptual framework that identifies transversal patterns, unresolved theoretical tensions, and generalizable design principles (Section 5 and Section 6). This structure supports the proposed three-dimensional analytical framework, which systematizes ionic selectivity at the convergence of transport mechanisms, dynamic structural states, and matrix complexity, providing a shared thermodynamic language developed in the subsequent sections. This approach relates progress in polymers of intrinsic microporosity, metal–organic frameworks, and ion-exchange membranes within a common thermodynamic language, providing a common design language for industrial applications.

Although recent studies have addressed metal ion recovery, selectivity remains vulnerable under operational stress, where conventional continuum transport models fail to predict pore expansions (up to 60%) [15]. The analysis identifies the collapse of the ionic cross-linking network and ionic strength-induced chain rearrangement as the primary drivers of the degradation of selectivity from 16.60 to 5.67 under industrial conditions [17]. The fixed-site hopping mechanism in hybrid PIM-ED systems is discussed as a possible explanation, a frontier often labeled as elusive or ambiguous, providing flux evidence to challenge current permeability–selectivity trade-off boundaries [18].

To situate the present review within the existing literature, Table 1 summarizes representative recent reviews on ion-separation membranes, highlighting their membrane families, the dominant transport principles discussed, and the gaps that remain unresolved. This comparison highlights the novelty of the present manuscript: the integration of five research streams into a unified three-dimensional analytical framework that connects transport mechanism, dynamic structural state, and system complexity.

In contrast, the present review brings together five research streams under a unified three-dimensional analytical framework that connects transport mechanism, dynamic structural state, and system complexity under real operating conditions. Table 1 therefore serves not as a complete bibliography of the field, but as a focused map of how the literature has approached the problem and why a broader, more integrative perspective is still needed. This positioning clarifies the niche of the present work before the framework is introduced in the following section.

**Figure 1:**
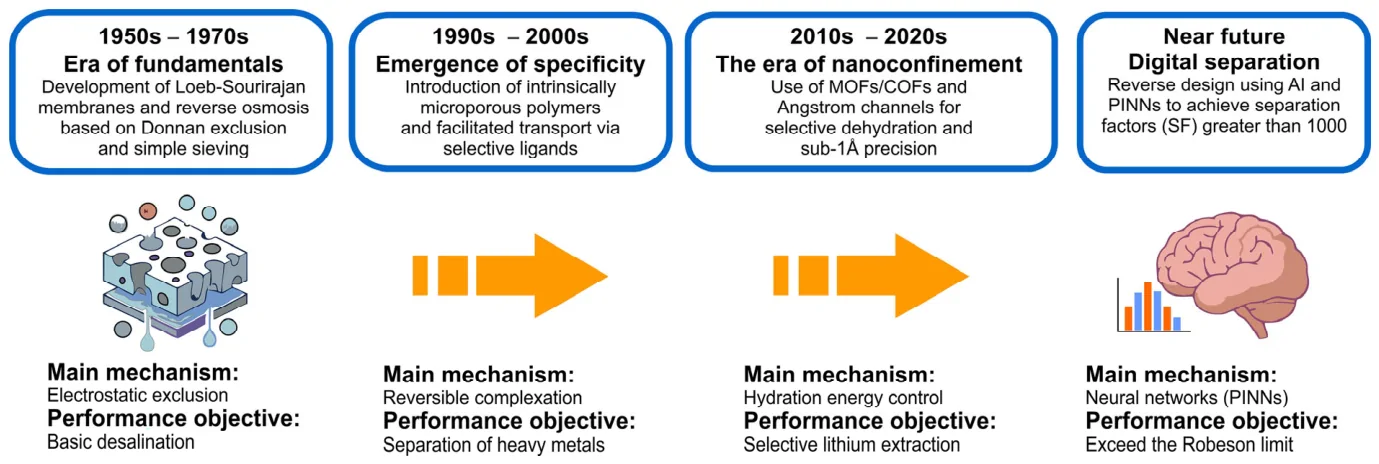
Evolutionary trajectory of polymeric membrane architectures toward single-species ion selectivity. The progression maps the shift from early macroscopic filtration, governed by electrostatic exclusion, to the manipulation of desolvation barriers within nanoconfined environments. The trajectory leads toward the digital separation era, where artificial intelligence (AI) and physics-informed neural networks (PINNs) support the inverse design of precision membranes.

To frame the historical development of the field, Figure 1 outlines the evolutionary trajectory of polymeric membrane architectures toward single-species ion selectivity. The figure traces the progression from early macroscopic filtration, dominated by electrostatic exclusion, to increasingly refined control over ion transport through nanoconfinement and desolvation-barrier manipulation. It then extends this trajectory toward the near-future digital separation era, in which AI and PINNs are expected to support the inverse design of precision membranes.

This framing is useful because it places the present review within a broader technological sequence rather than treating membrane selectivity as a static property. In that context, the three-dimensional framework developed later in the manuscript is presented as the analytical tool that connects this historical evolution with transport mechanism, dynamic structural state, and system complexity under real operating conditions.

## 2. Review Methodology

### 2.1. Search Strategy and Database Coverage

To ensure comprehensive thematic coverage, the scientific literature was screened and selected using a protocol tailored to subnanometric transport and polymeric architectures. The systematic search was completed on 15 August 2026, across three academic databases: Clarivate Web of Science, Scopus, and Google Scholar. Database-specific queries were conducted independently to mitigate indexing gaps, prioritizing Clarivate Web of Science and Scopus for peer-reviewed primary research. Google Scholar was used as a complementary source for foundational and historical publications.

The database-specific Boolean search string utilized for Clarivate Web of Science and Scopus was defined as follows: TS = (“polymer* membrane*” OR “ion-exchange membrane*” OR “polymer inclusion membrane*”) AND (“metal ion*” OR “lithium” OR “heavy metal*”) AND (“selective*” OR “transport*” OR “separation*”). Searches were limited to Title, Abstract, and Author Keywords. No initial geographic or institutional restrictions were applied.

### 2.2. Inclusion and Exclusion Criteria

The inclusion criteria were limited to peer-reviewed articles and reviews published within a ten-year window spanning from 2017 to 2026. This specific timeframe covers the contemporary state of the field, recognizing that the fundamental molecular and computational paradigms of sub-angstrom ion transport advanced considerably during this period.

Foundational references published before 2017 were retained and treated as exceptions when they were required to establish historical and theoretical baselines. These exceptions include: (a) seminal formulations of the Donnan equilibrium framework applied to ion-exchange membranes, (b) early empirical quantifications of the trade-off between membrane permeability and selectivity, and (c) early systematic characterizations of transport mechanisms in polymer inclusion membranes.

Studies focusing on conventional non-polymeric dense inorganic membranes (such as dense silica, metal oxides, and traditional zeolites) were excluded. However, porous crystalline framework membranes, specifically pure covalent organic frameworks (COFs), which are crystalline organic polymers, and metal–organic frameworks (MOFs), were included. This inclusion is justified because standalone framework architectures provide atomic-level criteria for evaluating the fundamental limits of sub-nanometric ion-sieving mechanisms and for informing the design of hybrid polymeric and mixed-matrix configurations.

### 2.3. Literature Screening and Source Tally

**Figure 2:**
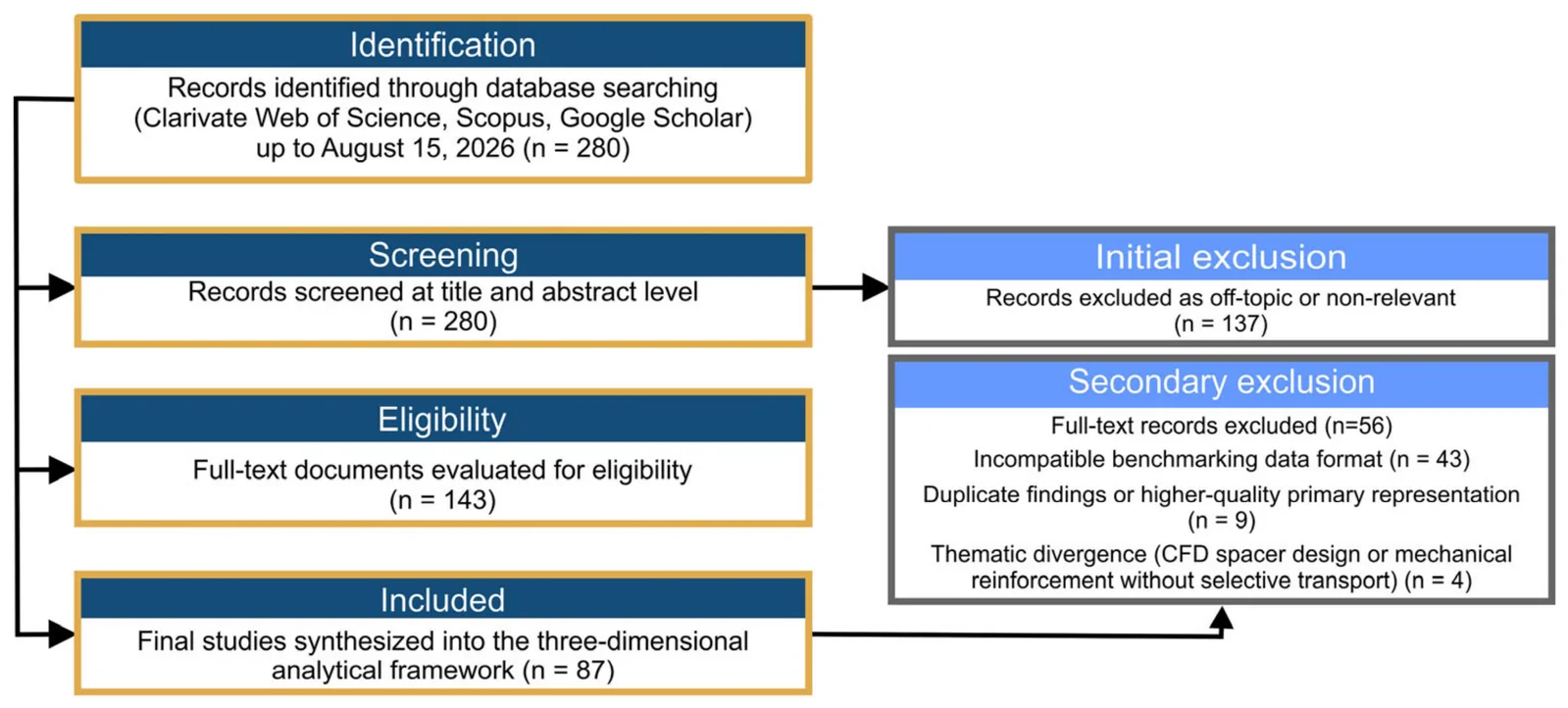
PRISMA 2020 flow diagram of the literature screening and selection process. A total of 280 candidate records were identified across three academic databases, 143 records were assessed in full text, 56 records were excluded at the full-text stage, and 87 sources were retained for synthesis and mapped onto the three-dimensional analytical framework.

Candidate records were screened in two stages (Figure 2). Duplicate records were first identified using Mendeley’s duplicate-detection function, and potential duplicates were subsequently verified and merged through manual cross-checking of bibliographic fields. In the first stage, titles and abstracts of the 280 candidate records were evaluated against the predefined inclusion criteria. Discrepancies at this level were resolved through consensus among authors. This initial screening resulted in the exclusion of 137 off-topic records, narrowing the pool to 143 records for full-text eligibility assessment.

In the second stage, each of these 143 records was evaluated in full. A total of 56 records were excluded at this full-text level for the following specific reasons: (a) 43 records lacked standardized quantitative benchmarking data, such as missing explicit driving forces, transmembrane flux values, or defined operating temperatures, which rendered them incompatible with our benchmarking protocol, (b) 9 reports presented duplicate findings or represented datasets already fully captured by higher-quality primary sources retained in the final selection, and (c) 4 records were excluded due to thematic divergence, focusing strictly on system-scale hydrodynamic spacer design via computational fluid dynamics (CFD) or mechanical reinforcement of polymeric supports without reporting species-specific ion transport selectivities.

Following this systematic screening cascade, the remaining 87 sources were retained for synthesis and organized into the three-dimensional analytical framework.

### 2.4. Synthesis Strategy and Framework Construction

The 78 sources were mapped onto the three analytical dimensions of the framework: mechanism, dynamic structural state, and matrix complexity. Sources were first grouped by the research stream they primarily represented, as developed in Section 3 and Section 4, and were then cross-referenced across dimensions to identify transversal patterns, theoretical conflict, and performance data suitable for benchmarking in Table 2, Table 3, Table 4 and Table 5.

Theoretical conflicts between sources were mapped as open divergences, as described in Section 3.2 and Section 4.2 and Section 5.2. This approach reflects the current stage of field development. Several fundamental mechanistic questions, including the dominant transport model in PIMs and the predictive validity of continuum models for subnanometric channels, remain unresolved in the primary literature, and the contribution of this review is to map those divergences rather than prematurely resolve them.

Quantitative data were compared only when the reported experimental conditions were sufficiently similar. When comparability was limited, the original operating conditions were specified in the corresponding tables. This practice reflects a core argument of the review: separation factor values have limited cross-study comparability when operating conditions are not specified precisely.

## 3. Mechanistic Foundations

Ionic selectivity in polymeric membranes cannot be attributed to a single physical or chemical principle. Instead, it emerges from the interaction between the molecular architecture of the membrane, the hydration state of its transport channels under real operating conditions, and the physicochemical properties of the competing ions. This interaction establishes the basis for rational membrane design; in the absence of a unified mechanistic framework, experimental selectivity data remain constrained to the specific system in which they were obtained [7,25]. This section establishes this framework at two levels. Section 3.1 describes the three fundamental mechanisms of ionic selectivity in polymeric membranes and identifies their internal tensions, specifically the conditions under which each mechanism becomes insufficient on its own. Section 3.2 examines the theoretical divergence between steric control and chemical control of selectivity, presenting evidence that this dichotomy requires an integrated treatment in which the dynamic state of the membrane plays a determining role.

### 3.1. The Three-Mechanism Framework and Its Internal Tensions

Selective transport of metal ions through polymeric membranes is governed by multiple mechanisms. Understanding these mechanisms individually is necessary, but it is not sufficient for rational membrane design, as their effects overlap, modulate each other, and in certain operating regimes, counteract one another. The following discussion presents them in order of increasing specificity, from global electrostatic control to the molecular recognition of an individual ion, as this ordering reflects the historical progression of the field and identifies where selectivity improvements remain underexplored.

An important point is that the membrane structure under operating conditions differs from its dry-state structure. Recent research demonstrates that ionic transport is governed by the reconfiguration of the micropore upon hydration, a process that substantially changes the geometry of the transport channels [13]. In the hydrated state, bound water mediates between electrostatic Donnan exclusion and steric sieving, as it alters the dehydration energy required for ions to access the pores [30].

The stability of cationic selectivity depends on the integrity of the ionic cross-linking network under continuous flow. In applications with real high-salinity brines (TDS of 409 g/L), it has been observed that the accumulation of metal cations at the polymeric interface triggers a rearrangement of polyethylenimine (PEI) chains, increasing the average intermolecular distance from 6.3 to 7.1 Å under intermediate salinity conditions, and reaching displacements of up to 22.9 Å in the charge centers of the chains under extreme saline stress [17]. This structural plasticity reduces monovalent/divalent selectivity under prolonged operation (e.g., K^+^/Mg^2+^) from initial values of 16.60 to 5.67 after prolonged operation periods. This phenomenon indicates that cationic selectivity is a dynamic state that collapses when electrostatic interactions are screened by the ionic strength of the medium, and that structural longevity is a design parameter as relevant as initial permeability [17].

Current continuum models have limited ability to describe ionic discrimination in angstrom-scale channels. While the solution–diffusion model assumes a homogeneous phase, Transition State Theory (TST) describes transport as a sequence of discrete barriers where selectivity emerges during dehydration partitioning [14,25]. The magnitude of this barrier is dictated by the hydration enthalpy of the cation; for example, species with high charge density, such as Mg^2+^ (−1830 kJ/mol), face a large energy penalty when attempting to enter confined pores, compared to Li^+^ (−475 kJ/mol) or K^+^ (−295 kJ/mol) [14]. This disparity creates a thermodynamic tension, because to achieve high flux, the membrane must provide coordination sites for compensatory interactions that lower the desolvation barrier, enabling ion transport in a partially dehydrated state [25,27].

Selectivity is therefore not a fixed property of the dry polymer, but a dynamic state that responds to the interaction between molecular architecture, the degree of hydration, and the operating conditions of the system. Table 2 provides a quantitative basis for comparing steric-hydration sieving with compensatory interaction. The data indicate that high SF values cannot be attributed solely to hydrated-radius discrimination; instead, they reflect how a given architecture modulates the desolvation barrier and the associated transport energetics. This chemical tuning mitigates the desolvation energy penalty, which reaches −1830 kJ/mol for Mg^2+^ compared to −475 kJ/mol for Li^+^ [17,31,32]. Consequently, architectures designed with coordination sites that replace the ion’s hydration shell alter the permeability–selectivity correlation documented in industrial matrices [33].

**Table 2:** Ionic thermodynamic properties and separation performance across representative polymeric membrane architectures.

Ion Pair (i/j)	ΔG_hyd_ (kJ/mol)	Δr_hyd_ (Å)	Membrane Architecture	Driving Force/Operating Mode	SF (i/j)	Dominant Mechanism	Ref.
Li^+^/Mg^2+^	1355	0.46	Standard IEM	Passive Diffusion (DD) or Electrodialysis (ED)	~5.0	Donnan Exclusion	[33]
Li^+^/Mg^2+^	1355	0.46	PSS@HKUST-1 (MOF)	Active Electric Field (Selective Electrodialysis)	1815	Electrostatic gating	[11]
Li^+^/Mg^2+^	1355	0.46	PIM-DB18C6-TB (Crown ether-TB)	Passive Diffusion (Diffusion Dialysis)	35.8	Host–guest recognition	[34]
Li^+^/Mg^2+^	1355	0.46	TPA100 (Crosslinked TB)	Passive Diffusion (Diffusion Dialysis)	157	Angstrom-scale size sieving	[35]
Li^+^/Mg^2+^	1355	0.46	CAIP-MOF (Charge-mosaic)	Pressure-driven Nanofiltration (NF)	108	Locally enhanced Donnan	[36]
K^+^/Mg^2+^	1535	0.97	PEI/PS-DVB (Real Brine)	Active Electric Field (Electrodialysis)	5.67	Structural plasticity (Failure)	[17]
K^+^/Mg^2+^	1535	0.97	UiO-66-OMe (MOF)	Passive Diffusion (Diffusion Dialysis)	1567	Compensatory interaction	[32]
K^+^/Mg^2+^	1535	0.97	TPA100 (Crosslinked TB)	Passive Diffusion (Diffusion Dialysis)	249	Hydration-size sieving	[35]
Cu^2+^/Ni^2+^	30	0.15	ZIOS (Adsorptive)	Passive Adsorption/Coordination Kinetics	~200	Molecular recognition	[31]
Li^+^/Na^+^	45	0.24	12-Crown-4 Polymer	Passive Diffusion (Diffusion	2.3	Facilitated transport	[37]
Bi^3+^/Cu^2+^	N/A	N/A	PFOAM50 (Amine)	Electro-assisted Dialysis (PIM-ED Mode)	471.4	PIM-ED Synergy	[18]

#### 3.1.1. Electrostatic Control via Donnan Exclusion (Mechanism 1)

Membranes bearing fixed charged groups generate a Donnan potential that discriminates ions according to their electric charge. Cation exchange membranes are used to favor the passage of cations while excluding co-anions, and anion exchange membranes operate in a complementary manner. This mechanism has been well characterized theoretically and forms the functional basis for conventional ion-exchange membranes and electrodialysis systems with decades of industrial application [38,39]. Within these charged polymeric networks, this classical thermodynamic phenomenon is governed by the Donnan potential at the membrane interface, which has been recently modeled for complex multi-ion solutions to determine the boundary limits of separation effectiveness (separation effectiveness of ideal ion exchange membranes, application of the Gibbs–Donnan theory).

Classical Donnan exclusion is insufficient to explain selectivity between ions of the same charge, such as Cu^2+^ versus Zn^2+^ or Pb^2+^ versus Cd^2+^, as the theory predicts only a general preference for ions of higher valence without discrimination between ions of the same group. Experimental data show a more complex picture. Ozkul et al. [40] demonstrated that in multi-ionic electrodialysis systems, the mechanisms of diffusion, electromigration, and convection operate simultaneously with varying contributions, such that the observed selectivity cannot be reduced to a single electrostatic exclusion principle. This limitation defines the theoretical horizon of the mechanism and motivates the development of the following two mechanisms.

#### 3.1.2. Steric Sieving and Hydration Thermodynamics (Mechanism 2)

Size-based discrimination becomes relevant because ions in solution do not exist as bare spheres but as hydrated complexes whose effective radius depends on the specific hydration energy of each ion. Two ions with similar bare ionic radii can have notably different hydrated radii, opening the possibility of discriminating between them using appropriately controlled pore sizes.

Selective ion transport through polymeric micropores has been shown to depend not on dry-state pore size but on the hydrated pore structure [13], which changes the geometry of the transport channels. In highly charged polymeric matrices, this confinement-driven transport is profoundly regulated by the state of bound water within the channels, which acts as a thermodynamic mediator that restricts co-ion permeation and enhances species-specific selectivity [41]. Controlling the hydrophobicity of pendant groups within the micropore determines the degree of hydration of the channel and, consequently, the effective transport radius under real operating conditions. Therefore, optimization of the dry porous structure without accounting for hydration changes leads to deviations in operational selectivity predictions. In practical terms, the actual control parameter is not the geometry of the synthesized pore but the structure of the hydrated pore under target process conditions.

#### 3.1.3. Specific Chemical Affinity and Compensatory Interactions (Mechanism 3)

In PIMs, a carrier embedded in the polymeric matrix forms reversible complexes with specific metal ions. This mechanism operates under thermodynamic and kinetic principles that differ from those of the previous two mechanisms, as selectivity does not depend on pore size or the fixed charge of the membrane but on the stability constant of the ion–carrier complex and the kinetics of complexation and decomplexation at the membrane-solution interfaces [24].

This mechanism can offer greater specificity than the previous two, given that the carrier can be designed so its coordination geometry and chemical affinity match those of a target ion. However, this specificity is limited by factors that electrostatic and steric mechanisms do not share to the same extent, including carrier stability within the matrix under prolonged operating conditions, transport kinetics that may be limiting at low carrier concentrations, and the sensitivity of the complex stability constant to process variables such as pH and temperature [8].

The three mechanisms described above are not independent variables but axes of a shared design space. Their integration into a Three-Dimensional Analytical Framework is developed in Section 5.3 based on the comparative evidence presented across Section 3 and Section 4.

### 3.2. Steric Control vs. Chemical Control

Ionic selectivity is often classified either as a physical phenomenon or as a chemical interaction. This binary formulation is useful for framing the debate, but recent evidence suggests that the dichotomy is a simplification.

Liu et al. [25] analyzed ion–ion selectivity in synthetic membranes with confined nanostructures and concluded that the molecular mechanisms of ionic transport under nanometric confinement simultaneously integrate steric, electrostatic, and specific interaction effects, with the relative dominance of each mechanism depending critically on the scale of confinement and solution conditions. This integrative framework surpasses previous one-dimensional perspectives but leaves open the question of how to predict a priori which mechanism will dominate in a given design system, which is precisely the most relevant rational design question.

Robeson [7] conducted a systematic evaluation of membrane materials for selective ion separations and identified that conventional polymeric membranes show limited ion–ion selectivity due to the heterogeneity of their free volume elements. According to these authors, progress requires either precise molecular control over these elements or the incorporation of specifically designed ion-membrane interactions, suggesting that neither a purely steric nor a purely chemical approach is sufficient on its own. Evidence from COF-based membranes supports this view, as despite the appeal of their well-defined crystalline pore geometries, claims of ordered structure and discrimination mechanisms remain contested, and reported selectivity data frequently resist reproduction outside the original synthesis conditions [42].

Espinoza et al. [30] showed that bound water in highly charged polymeric membranes modifies ionic selectivity by acting as a mediator between electrostatic and steric mechanisms. This finding introduces the state of water within the membrane, and not just the polymer structure, as a determining variable for selectivity. This finding suggests that the composition and hydration state of the membrane under operation are parameters that must be explicitly characterized, rather than inferred from the dry-state structure. Taken together, these works support a conclusion developed throughout the review, since ionic selectivity in polymeric membranes is an emergent phenomenon of the entire system, not an additive property of its components. This conclusion has direct consequences for the way characterization experiments are designed, flux and selectivity data are interpreted, and laboratory results are transferred to real operating conditions.

The transition from steric to chemical control has been associated with single-species selectivity. Pure steric control, based on the Stokes radius, is ineffective for pairs with similar hydrated radii; however, chemical tuning of the pore wall allows for the selective manipulation of the activation energy (Ea) of transport [27]. Work on UiO-66 channels shows that by modifying functional groups from −OH to −OMe, it is possible to broaden the activation energy difference (ΔEa) between the target cation and the interferent (e.g., from 17.1 to 22.2 kJ/mol for the K^+^/Mg^2+^ pair) [32]. These results show that chemical control, through the regulation of ionic affinity and repetitive dehydration in sub-nanometric windows, yields selectivities exceeding SF 1000, which simple geometric sieving cannot sustain under real flow conditions [33]. The rational translation of this principle into designed molecular systems requires a system-level design methodology that integrates thermodynamic descriptors, synthesis constraints, and process boundary conditions from the outset. Molecular system design frameworks that formalize these multi-objective requirements provide a basis for translating mechanistic understanding into quantitative design rules [43].

## 4. Five Research Streams and Their Convergences

The field of polymeric membranes for selective metal-ion separation is structured into five research streams. These streams are not mutually exclusive or chronologically successive because they coexist, partially overlap, and in some cases converge toward hybrid strategies. The streams are presented here in order of mechanistic maturity, moving from systems with a more established theoretical base to those of greater novelty and lower operational maturity, as this ordering allows a clearer mapping of the theoretical tensions analyzed in Section 5. For each stream, the dominant selectivity mechanism, the consensus reached in the recent literature, unresolved divergences, and the conditions under which the reported results are transferable to other systems are identified.

**Figure 3:**
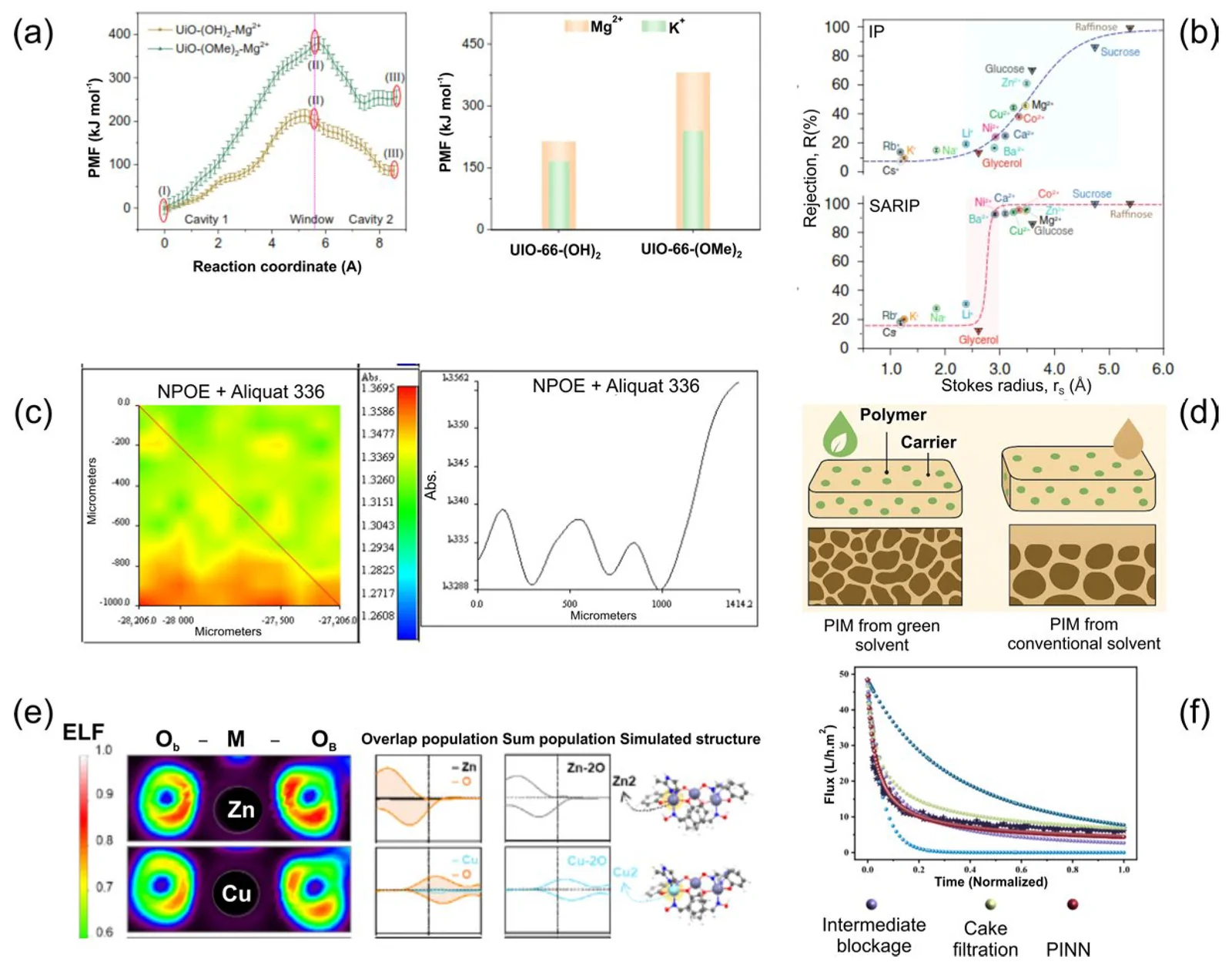
Literature-based comparison of representative membrane systems, compiled from recent studies on thermodynamic barriers, microstructural characterization, and predictive modeling: (**a**) potential of mean force free energy barriers for cation transport, reproduced from Mo et al. (2024) [32]; (**b**) pore size distribution curves, adapted from Liang et al. (2020) [44]; (**c**) Raman imaging microscope (RIMM) cross-sectional maps, adapted from Macías and Rodríguez de San Miguel (2023) [45]; (**d**) schematic comparison of polymer inclusion membrane morphology, adapted from Nowik-Zając and Sabadash (2025) [8]; (**e**) thermodynamic transmetalation energies correlated with Bader charge differences, adapted from Le et al. (2024) [31]; (**f**) representative physics-informed neural network (PINN) flux-decline prediction, adapted from Garakani et al. (2026) [46]. The figure is intended as a qualitative cross-study synthesis rather than a universal criterion.

To contextualize the performance of the architectures discussed in this section, Figure 3 presents a literature-based comparison of separation factor and lithium flux for representative membrane systems. The figure is meant as a qualitative synthesis of reported trends across membrane classes, not as a universal benchmark, because the underlying studies were conducted under different driving forces, feed compositions, and operating conditions. In this sense, the plot helps locate the apparent balance between selectivity and productivity for each system, while preserving the interpretive limits imposed by cross-study heterogeneity.

### 4.1. Polymer Inclusion Membranes (PIMs) and Electromembranes

PIMs represent the most active research stream in the analyzed literature and serve as a representative case of design based on chemical selectivity. A typical PIM is composed of three elements: a base polymer, frequently cellulose triacetate (CTA) or polyvinyl chloride (PVC), a plasticizer that confers mechanical flexibility, and an extractant carrier that determines selectivity toward specific metal ions. Consequently, modifying PIM architectures involves changing from passive diffusive transport to electro-assisted mechanisms. The transition from passive to electro-assisted transport in PIM-ED systems, whose quantitative performance evidence is developed in Section 4.2.2, represents the most relevant recent advancement in this stream [47].

#### 4.1.1. Consensus Reached

PIMs have been reported to exhibit higher selectivities than conventional IEMs when discriminating between metal ions of similar charge. This mechanistic superiority is based on carrier-mediated facilitated transport, where ion–carrier coordination chemistry and host–guest interactions replace the non-specific Donnan exclusion and steric sieving typical of conventional IEMs [48,49]. While standard IEMs suffer from electrostatic charge screening under high ionic strength, PIMs maintain species-specific recognition via physically entrapped active carriers, such as task-specific reactive ionic liquids [50,51] or custom-synthesized alkylimidazole derivatives [48], which form stable coordination complexes with target metal ions within the polymeric matrix. Wang et al. [24] reported that PIMs have demonstrated selective transport capacity for a wide range of metal ions, from transition metals to rare earths and precious metals, with separation factors exceeding several orders of magnitude. Wang et al. [52] proposed a dual PIM system for the simultaneous separation of Cu^2+^, Zn^2+^, and Mg^2+^, demonstrating that the combination of two different carriers (LIX 84-I and D2EHPA) in separate membranes allows for selectivities that a single carrier cannot achieve.

Furthermore, recent structural and morphological analyses showed that the separation kinetics and flux of PIMs are tunable through the selection of the polymer matrix (e.g., hydrophobic PVC versus hydrophilic CTA) and the addition of specific plasticizers [53,54]. For instance, the incorporation of phosphonium-based ionic liquids in CTA matrices yields more hydrophilic, amorphous, and rougher surfaces compared to PVC, enhancing metal-ion accessibility to the internal coordination sites [54]. Additionally, plasticization-induced oriented micro-channels within the polymer matrix can lower the transport barrier, facilitating rapid diffusion [55], while the mechanical and morphological properties (governed by the plasticizer-to-carrier ratio and mechanical stretching) dictate the physical pathways available for metal-ion pertraction [53].

Hernández-Fernández et al. [51] showed that PVC membranes containing methyltrioctylammonium chloride ([MTOA^+^][Cl^−^]) achieved Zn^2+^/Cu^2+^ separation factors of 1996 and Zn^2+^/Fe^3+^ factors of 606. These values illustrate the potential of chemical design while simultaneously raising questions regarding the conditions under which such selectivities are maintained, particularly under competitive, high-salinity multi-ionic matrices, such as real seawater, where conventional IEM selectivities typically collapse [45]. To transition these high-selectivity designs toward industrial applications, recent efforts have focused on sustainable fabrication without compromising selectivity. This includes the formulation of task-specific synergistic systems: such as [A336][P507] ionic liquids embedded in hydrophilic copolymers for adjacent rare-earth separation (Lu/Gd/Yb) [56], and the substitution of toxic chlorinated solvents with green alternatives like ethyl acetate or 2-methyltetrahydrofuran [57]. PIMs prepared with green solvents exhibit comparable extraction efficiencies and physical stability to conventional systems [57], demonstrating that environmentally friendly production is highly compatible with high-precision separations [24].

#### 4.1.2. Unresolved Divergences and Tensions

A core divergence in the PIM field lies in the transport mechanism of the carrier within the polymeric matrix. There are two competing models: a) the mobile carrier model, which postulates that the extractant diffuses freely through the membrane, forming and breaking complexes at the interfaces, and b) the fixed-site jumping model, which proposes that ion–carrier complexes are transferred between relatively stationary carrier sites within the matrix. Wang et al. [24] noted that a deeper understanding of membrane microstructure and separation mechanisms is needed to advance the field. Resolving this controversy has direct implications for design, as the diffusivity of the carrier in the matrix determines flux if the mobile carrier model is correct, whereas the density and spatial distribution of carrier sites are the key design parameters if the jumping model dominates.

Long-term stability remains a persistent problem. Zioui et al. [58] studied the durability of PIMs based on PVDF/CTA with D2EHPA as a carrier for the competitive transport of Ni^2+^, Pb^2+^, and Zn^2+^, observing a selectivity order of Ni^2+^ (45%) > Pb^2+^ (35%) > Zn^2+^ (5%). Nowik-Zając and Sabadash [8] noted that performance degradation when PIMs are exposed to real conditions, such as pH fluctuations, temperature, and the presence of organic matter, is a barrier to industrial adoption. Hernández-Fernández et al. [51] found that membranes based on omim^+^PF6^−^/PVC in a 30/70 ratio retained up to 82% of the initial ionic liquid weight after 2048 h (85 days) of exposure to a metallic solution in 1 M HCl, while MTOACl/PVC (70/30) membranes only retained 48%. This progressive loss of carrier compromises selectivity and operational viability.

Macías and Rodríguez de San Miguel [45] extended the application of PIMs to the selective separation of Pb^2+^, Cd^2+^, and Zn^2+^ from seawater, demonstrating that the complexity of real matrices, including high ionic strength and the presence of multiple competing cations and anions, significantly alters separation factors compared to synthetic solutions.

Resolving this ambiguity through in situ experimental characterization and multi-scale computational modeling is one of the five priority research questions developed in Section 6.

### 4.2. Ion-Exchange Membranes and Electrodialysis

IEMs, including cation exchange (CEMs) and anion exchange (AEMs) varieties are the most industrially mature membrane technology for the treatment of electrolytic solutions. Their selectivity principle is electrostatic, i.e., fixed charged groups in the polymeric matrix attract counterions and repel co-ions through the Donnan exclusion mechanism. This generates a membrane potential that discriminates ions based on their charge and, to a lesser extent, their mobility within the membrane phase [19,38]. When integrated into electrodialysis (ED) systems, the electrical driving force amplifies transport and allows for continuous operation with high productivity. However, selectivity between ions of identical charge, such as Li^+^ versus Na^+^ or Mg^2+^, or Cu^2+^ versus Zn^2+^, cannot be explained by classical Donnan exclusion, which is the fundamental mechanistic limit of this membrane family and the central problem in its recent development [40,59,60].

Overcoming the selectivity limit for cations with identical valences requires the design of specific coordination sites that discriminate by thermodynamic affinity rather than net charge. In systems using iminodiacetic acid (IDA) groups, density functional theory (DFT) calculations have shown that binding energy follows the Irving–Williams order (Cu^2+^ > Ni^2+^ > Zn^2+^ > Co^2+^ > Mg^2+^), reaching values up to −543 kJ/mol for Cu^2+^ compared to only −146 kJ/mol for Mg^2+^ [12]. This disparity allows ultra-thin polyelectrolyte membranes to achieve Cu^2+^/Ni^2+^ separation factors near 30 in bi-cationic solutions, as the flux of strong-binding species depends on thickness while that of weak species is independent, enabling selective permeability based on ligand exchange kinetics [12]. Recent advances have introduced mechanically reinforced GO-COF composite membranes with a nacre-inspired architecture, achieving monovalent/divalent selectivity (up to 15.34 for Na^+^/Ca^2+^) while maintaining high charge efficiency in synthetic hypersaline environments [61].

#### 4.2.1. Advances in Mechanistic Understanding

In ED systems, the apparent selectivity of IEMs is evaluated as an emergent property rather than an intrinsic material property. Ozkul et al. [40] analyzed the relative contributions of diffusion, electromigration, and convection on ionic selectivity in multi-ionic solutions during ED, demonstrating that operating conditions such as current density, feed concentration, or ionic composition alter which mechanism dominates transport at any given time. This multi-mechanistic perspective goes beyond simplified formulations based exclusively on Donnan exclusion. It has direct implications for process design, as selectivity optimization cannot be performed solely at the material level but requires co-optimization of the material and operating conditions.

In ED, selectivity should be regarded as a dynamic process-level property that evolves with operating conditions, including current density, feed composition, and salinity. Likewise, PIM-based systems exhibit time-dependent performance governed by carrier retention, matrix stability, and the interaction between the electric field and the carrier-mediated transport pathway.

A relevant parallel advancement concerns monodivalent selectivity, which is one of the most practically relevant problems in the field for both recovering valuable monovalent metal ions from complex matrices and selectively removing divalent contaminants in the presence of background electrolytes. Rijnaarts et al. [62] demonstrated that the surface modification of CEMs with polyelectrolyte layers could improve Li^+^/Mg^2+^ selectivity by introducing a surface steric sieving mechanism superimposed on the membrane’s electrostatic exclusion, providing an early example of mechanistic hybridization within the same membrane family. More recent developments have extended this strategy by designing IEMs with functional groups specific to monovalent ions, achieving Li^+^/Mg^2+^ separation factors that compete with those of adsorption systems [17,63].

The operational limitations of IEMs at high current density have been characterized with greater precision in the recent literature. Beyond the limiting current density, concentration polarization at the solution-membrane interface and water dissociation introduce effects that degrade both separation performance and the chemical integrity of the membrane’s functional groups. Gurreri et al. [64] modeled and experimentally validated these effects in pilot-scale electrodialysis systems, establishing that ED module design must explicitly incorporate longitudinal concentration profiles and boundary layer phenomena to predict real selectivity under process conditions, a dimension frequently ignored in laboratory studies at the small-cell scale.

The current consensus in ED has evolved from a homogeneous phase view toward the analysis of discrete energy barriers in charged pores. It has been identified that conventional IEMs face a degradation of permselectivity in high-salinity conditions due to surface charge screening, which reduces the efficacy of Donnan exclusion [37]. To counteract this, it has been shown that the control of bound water is a key dynamic mediator, as in membranes with high charge density, the transition of water to a non-freezing state enhances both partitioning and diffusion selectivity, allowing the basal transport limit to be exceeded in hypersaline brines [31].

#### 4.2.2. PIM-ED Coupling as a Mechanistic Hybridization Strategy

In this configuration, the applied electric field primarily accelerates carrier-mediated transport by driving the ion–carrier complex toward the membrane interface and facilitating interfacial complexation/decomplexation kinetics. Selective transport then proceeds through a fixed-site jumping mechanism within interconnected microphase channels, rather than through electro-osmotic solvent drag alone. The integration of PIMs into electrodialysis (PIM-ED) processes has opened routes for the selective recovery of high-value metals such as Bi^3+^ and rare earths [2,31]. Using tertiary amines (trioctylamine) as immobilized carriers, Bi^3+^ fluxes of 131 × 10^−2^ mmol m^−2^ h^−1^ have been achieved, exceeding the performance of standard commercial membranes by 5.3 times [18]. In these systems, metallic transport occurs through a fixed-site jumping mechanism, where the electric field accelerates transfer kinetics through interconnected microphase channels [18]. For transition metals such as Zn^2+^ and Fe^3+^, the use of quaternary ammonium-based ionic liquids ([MTOA^+^][Cl^−^]) allows for separation factors superior to 1900 for the Zn^2+^/Cu^2+^ pair, demonstrating that selectivity can be tuned through the IL/polymer ratio to optimize the intrinsic mobility of the metal–carrier complex [51]. This hybridization mechanism is governed by a fixed-site jumping phenomenon that activates upon exceeding critical current density thresholds (approx. 7 mA/cm^2^), thereby transforming slow diffusive transport into a high-ionic-conductivity pathway.

Results illustrating the potential of this strategy were reported by Wang et al. [18]. They showed that polymeric membranes functionalized with trioctylamine as a carrier, manufactured by pore-filling solvent casting, achieved high permselectivities in ED systems for Bi^3+^ separation, compared with reference commercial membranes, in the presence of multiple competing metal ions. These results, if confirmed, suggest that mechanistic hybridization can shift the flux–selectivity trade-off boundary beyond what is achievable by each individual approach, opening a design pathway developed conceptually in Section 4.3. However, available data correspond to laboratory conditions with synthetic solutions, and the stability of the PIM-ED system under sustained operating conditions has not yet been demonstrated.

The convergence between IEM-ED and PIMs represented by the PIM-ED system points to a direction that goes beyond incremental optimization within each stream, as the architecture of the coupling between mechanisms, rather than just material selection, emerges as a first-order design parameter, a principle that the conceptual framework of Section 5.3 formalizes.

### 4.3. Nanostructured Membranes and Subnanometric Confinement

The research streams described in Section 4.1 and Section 4.2 share a common structural limitation. In PIMs, the carrier is distributed statistically in an amorphous matrix; in conventional IEMs, fixed charged groups exhibit a non-uniform distribution of density and accessibility. This heterogeneity imposes a ceiling on ion–ion selectivity between species of similar properties because real transport channels encompass a distribution of sizes, charges, and chemical environments that average and dilute molecular discrimination [7]. The third research stream addresses this limitation, seeking to define the geometry and chemistry of transport channels with subnanometric precision so that discrimination is a deterministic property of the individual pore rather than a statistical average.

#### 4.3.1. Consensus in the Recent Literature

Data in this research field indicate that discriminating between ions of equal charge requires simultaneous control of three parameters at the subnanometric scale: (a) the effective size of the channel in a hydrated state, (b) the density and spatial distribution of functional groups on the pore walls, and (c) the partial desolvation energy that the ion must overcome to enter the channel [13,25]. COF-based membranes represent a structurally distinct approach to this three-parameter control problem. Their crystalline lattices define pore windows with sub-angstrom precision and allow for post-synthetic functionalization of the pore walls. However, an evaluation of COF membranes in water treatment has identified that the correspondence between crystallographic pore geometry and actual transport selectivity in aqueous ion mixtures is frequently ambiguous, with membrane fabrication defects and intercrystalline voids generating non-selective transport pathways that dilute the ideal pore-level discrimination. This finding is consequential for the stream as a whole, indicating that crystalline pore definition is a necessary but not sufficient condition for high-resolution ion selectivity, since the overall uniformity of the membrane film determines whether ideal pore-level discrimination is maintained across the active area [42].

Modulation of the hydrophobicity of pendant groups has been shown to control the degree of hydration of the channel [13] and, consequently, the effective transport radius, which differs substantially from the pore radius in a dry state. Membranes with pendant groups of intermediate hydrophobicity achieved SF greater than 1000 between ion pairs of contrasting charge and hydrated radius in binary solutions, a result the authors attribute to the fine-tuning of differential desolvation energy within the hydrated channel.

A complementary strategy was adopted by Gu et al. [65] by basing subnanometric control on zwitterionic cyclodextrins assembled into membranes with uniform channel sizes. The uniformity of the channels, in contrast to the statistical distribution of free volumes in conventional polymeric membranes, allowed for efficient and selective removal of heavy metal ions, with consistent results across experiments that conventional heterogeneous membranes rarely reproduce. This work illustrates that channel uniformity is an advantage not only for performance but also for experimental reproducibility, a relevant criterion for industrial validation.

#### 4.3.2. Transferability Challenges Under Real Conditions

The primary divergence in this stream concerns the dominant mechanism of ionic discrimination within the defined pore, specifically whether it is steric sieving by hydrated pore size or specific ion-functional group interactions at the pore wall. Liu et al. [25] argued that under subnanometric confinement, both mechanisms operate simultaneously and their relative contributions depend on the scale of confinement and the ionic composition of the solution, an integrative response that nonetheless leaves open the question of how to predict a priori which mechanism will dominate in a given design system.

Morishita et al. [41] provided evidence that the spatial arrangement of functional groups on nanopore walls, not just their presence or density, modulates anionic selectivity. Membranes with zwitterionic groups in a specific spatial configuration showed discrimination between anions of similar charge, superior to membranes with the same density of groups but in a random configuration. This result introduces a design dimension that transport models based on averaged membrane properties cannot capture.

Zhou et al. [66] developed biselective microporous membranes based on Tröger’s base, capable of simultaneously separating cations and anions selectively, a result that challenges the conventional CEM/AEM dichotomy and suggests that the intrinsic porous architecture of certain polymers can generate multidimensional selectivities without the need for fixed charged groups. However, Tröger’s base systems present challenges in scalability and hydrolytic stability that limit their industrial projection in the short term, and reported separation factors correspond to synthetic solution conditions that have not been validated in complex matrices.

In nanofiltration systems, Tong et al. [15] provided a relevant operational perspective in which transmembrane pressure conditions actively modify the effective pore radius of piperazine membranes, with expansions of up to 60% relative to the static state, while simultaneously increasing the volumetric charge density by 4 to 5 times. This structural plasticity under operation has direct design implications. Under this condition, selectivity characterized in static or low-pressure conditions may underestimate or overestimate the real selectivity under process operating conditions, complicating the extrapolation of laboratory results to pilot or industrial scales.

Despite the success of sieving models, a divergence persists between basal permeability data and performance in real multicomponent mixtures. Studies in dipole-decorated channels reveal that electrostatic interactions between neighboring pores in dense membranes can suppress water flux and favor the passage of co-ions, challenging the assumption that membrane performance can be extrapolated from the behavior of a single pore [37]. This tension suggests that selectivity is not just a function of pore size but also of an emergent correlation between the pore network and the medium’s ionic strength. In addition, the development of smart architectures has introduced a new direction in dynamic selectivity. Specifically, bioinspired membranes functionalized with ion-recognizable microgels acting as stimuli-responsive gates have demonstrated the ability to regulate water flux and ionic transport through thermo- and ion-dual responsiveness, enabling the separation of trace Pb^2+^ ions [67].

#### 4.3.3. Systemic Limitations of the Stream

Despite the described advances, a mechanistically distinct strategy within the Tröger’s Base (TB) platform was reported by Dong et al. [34], who synthesized the PIM-DB18C6-TB membrane by incorporating dibenzo-18-crown-6 (DB18C6) units as Li^+^-selective functionality alongside TB units for free volume generation. In this architecture, selectivity is governed by host–guest recognition between the crown ether cavity and the Li^+^ ionic radius rather than by pore-size cutoff, yielding Li^+^/Mg^2+^ separation factors of 35.80 under static diffusion and 24.35 under electrodialysis. These values are lower than those reported for the more strongly size-constrained DMBP-QTB architecture (SF 152.2-−161.3), but the comparison should be interpreted cautiously because the reported systems differ in transport regime, membrane chemistry, and operating basis. The relevant conclusion is therefore not that chemical recognition is inherently weaker than steric-hydration sieving, but that the observed SF is highly system-dependent and cannot be ranked without matching the transport conditions. Accordingly, the apparent superiority of one mechanism over another should be treated as conditional on the ion pair, the membrane architecture, and the imposed driving force, rather than as a universal hierarchy of selectivity mechanisms.

More recently, Liu et al. [35] demonstrated that systematic tailoring of TB structural parameters, evaluated under variable Mg^2+^/Li^+^ ratios, pH gradients, and extended operation, improves mono/divalent selectivity while partially addressing the stability gap that remains unresolved in most TB architectures.

However, nanostructured membranes face three systemic limitations that condition their technological maturity. First, the manufacture of subnanometric pores with sufficient uniformity at a scale larger than a few square centimeters remains unsolved in process engineering [68]. Second, structural stability under prolonged exposure to real matrices, including pH variations, temperature, and competing ions, has not been demonstrated in any of the reported material families. Third, the synthesis cost of precursor materials, such as functionalized cyclodextrins, polymers of intrinsic microporosity, or TB polymers, is currently incompatible with high-volume applications, although this limitation is economic rather than mechanistic and could be resolved with the development of scalable synthesis routes.

These limitations do not undermine the theoretical value of the stream, but rather define the space separating current mechanistic knowledge from practical application, a theme developed in Section 4.5 and the integrative discussion of Section 5.

### 4.4. Modification Strategies and Composite Design

The previous streams develop ionic selectivity predominantly from a single mechanistic principle, such as carrier chemical recognition, electrostatic exclusion, or defined pore steric sieving, respectively. The fourth stream adopts a different logic; instead of optimizing an individual mechanism, it deliberately seeks to combine two or more selectivity mechanisms into a single material architecture so that their effects are complementary or mutually reinforcing. This composite design strategy combines materials synthesis with process parameters to address valuable ion recovery and toxic metal treatment [22,36].

#### 4.4.1. Inorganic-Polymeric Composite Membranes

The most documented case in the recent literature for this strategy is the separation of ions with different charges but similar bare ionic radii, where Donnan exclusion is insufficient, and the inorganic component provides the necessary additional discrimination. Saif et al. [69] developed composite membranes of sulfonated polyethersulfone (SPES) incorporating hydrogenated manganese oxide (HMO), a material with crystallographic cavities of a size related to the bare ionic radius of small monovalent cations, achieving a selective ionic conductivity of 8.28 mS/cm, thirteen times higher than the HMO-free control, and an ideal mono- and divalent cation selectivity of 11.75 through diffusion dialysis.

More recent developments have extended this principle to other material families; for example, zeolites with Li^+^ selectivity, carbon molecular sieves, and metal–organic frameworks (MOFs) with controlled pore sizes have been incorporated into polymeric matrices with promising selectivity results, although with persistent challenges regarding interfacial compatibility between the inorganic component and the organic matrix [70].

The interface between the inorganic component and the polymeric matrix is the most vulnerable point, as adhesion defects generate non-selective leakage channels that degrade the overall system selectivity. Lu and Wang noted that interfacial compatibility between the porous component and the organic matrix is one of the persistent challenges in this material family, and its resolution is a necessary condition for the structural selectivity of the inorganic component to be effectively expressed in the composite membrane [8].

When the transport channel size is reduced to the angstrom scale, ionic selectivity is governed by the energy barrier required for the partial dehydration of the cation [13,14]. Cations such as Li^+^, despite having a small bare ionic radius (0.60 Å), possess a high hydration enthalpy (−475 kJ/mol) compared to K^+^ (−295 kJ/mol), generating a significant entry barrier in confined pores [1,14]. This principle is exploited by MOF membranes such as ZIF-8, whose 3.4 Å windows force a selective desolvation that allows for discrimination between alkali metals [70]. In Li^+^/Mg^2+^ separation, fundamental for brine mining, the use of charged sub-nanopores allows for separation factors up to 284, as Mg^2+^ faces a much higher dehydration energy penalty (−1830 kJ/mol) that prevents it from effectively penetrating the hydrated channel [14]. Consequently, architectures capable of offering specific coordination sites can shift performance beyond conventional limits. One advancement in this direction involves metal–organic framework glass membranes with interconnected charged channels, which achieve Li^+^ permeation rates 60-fold higher than their crystalline counterparts while enhancing Li^+^/Mg^2+^ separation through a significantly higher energy barrier for magnesium desolvation [71].

#### 4.4.2. Surface Modification and Functionalization of Base Membranes

A second composite strategy is based on the surface modification of a base polymeric membrane, typically a commercial nanofiltration or ion-exchange membrane, through the deposition of thin functional layers that add a selectivity mechanism in addition to that of the underlying membrane. Siekierka et al. [22] comparatively analyzed improved separation mechanisms through polymeric modification, identifying that the combination of ion exchange, surface electrostatic interactions, and steric entrapment in a single membrane offers selectivity advantages over monomechanistic approaches, but at the cost of higher manufacturing complexity and lower batch-to-batch reproducibility.

Recent refinements have been achieved through layer-by-layer (LbL) deposition of polyelectrolytes with alternating charges on base membranes, generating a surface architecture with mono/divalent selectivity that can be tuned by controlling the number of layers and the charge density of each polyelectrolyte [59,72]. This design flexibility is a significant operational advantage, allowing for the adaptation of the selectivity of a standard commercial membrane without modifying its base manufacturing process, thereby reducing the scaling barrier compared to newly synthesized membranes.

#### 4.4.3. 2D Materials and Bioinspired Membranes

The most active area within this stream involves the incorporation of two-dimensional materials (graphene oxide, MXenes, hexagonal boron nitride, molybdenum disulfide). Recent advances in nanoarchitectonics have introduced localized assembly strategies that precisely orient functional groups within 2D channel environments, demonstrating that the spatial configuration of recognition sites, rather than their density alone, governs the selectivity limit of 2D membrane architectures [73]. The use of such 2D materials has enabled the construction of nanochannels with atomic thicknesses that minimize transport resistance [31]. Inspired by biological channels, membranes with concerted single-file transport have been developed, where multi-ion interaction reduces energy barriers (e.g., from 3.87 eV to only 0.15 eV for uranyl), allowing elevated fluxes with selectivities up to 734 against interferents such as vanadium [14]. Furthermore, the integration of crown ethers into MOF matrices has demonstrated the ability to force complete ion dehydration, reaching Li^+^/Mg^2+^ separation factors of 1815 under an electric field [37].

Selective ion transport through 2D confined spaces has been reviewed by Zhao et al. [68], identifying that angstrom channels formed between sheets of 2D materials offer ionic selectivities that rival those of biological protein channels under controlled conditions, but that long-term structural stability and selectivity degradation in complex matrices remain unresolved challenges for practical adoption.

Tonnah et al. [37] examined bioinspired and engineered ion-selective membranes, noting that the transition from laboratory demonstrations to real-scale applications requires demonstrating reliability under sustained operating conditions, a criterion that no system based on 2D materials has met to date. The gap between ideal laboratory performance and real operation is more pronounced in this material family. This is precisely because its selectivity mechanism depends on the integrity of angstrom-scale structures susceptible to water molecule intercalation, differential swelling, and structural reorganization under dynamic conditions.

For the separation of ions with similar physicochemical properties, 2D materials provide a relevant theoretical perspective. Angstrom channels formed between sheets of materials such as reduced graphene oxide allow for discrimination between ions with contrasting hydrated radii through combined sieving of ionic radius and desolvation energy, a mechanism relevant for both the recovery of valuable monovalent cations and the selective removal of divalent heavy metals in complex matrices [68]. However, selectivities demonstrated in laboratory conditions with synthetic solutions are reduced in the presence of competing ion concentrations characteristic of real industrial matrices, limiting the direct transferability of those results to process applications.

#### 4.4.4. Critical Evaluation of the Stream

Composite design is the stream with the greatest diversity of strategies and the widest dispersion of results. The combination of mechanisms can yield properties superior to those of each component, but it can also introduce complex failure modes. Interfacial degradation, selective loss of one component, and differential response to operating conditions are among the harder events to diagnose and correct compared to monomechanistic system failures. Siekierka et al. [22] noted that manufacturing reproducibility is the most frequent operational limitation in composite membranes, a problem that scales non-linearly as membrane area increases. This characteristic makes composite design more suitable, in its current state, for high-value-added niche applications, such as lithium recovery from concentrated brines, than for large-scale water treatment at a low unit cost.

To contextualize the efficacy of the various architectures presented, Table 3 presents the performance parameters for representative metal cations under different separation mechanisms. A disparity in SF is observed, where systems based on chemical coordination, such as the ZIOS platform, manage to discriminate divalent cations with SF values greater than 200 for the Cu^2+^/Ni^2+^ pair and up to 2049 for Cu^2+^/Mn^2+^ [31]. This contrasts with blending systems such as quaternized polymer of intrinsic microporosity with 15% functionalization (QPInM15)/SPEEK, whose values for Li^+^/Mg^2+^ (SF = 6.98) suggest that hybridization without strict subnanometric control still faces limitations in shifting the permeability–selectivity trade-off boundary [37]. Nevertheless, the case of PFOAM50 membranes in electrodialysis, with bismuth fluxes reported in Section 4.2.2, validates the thesis that applying an external electric field can accelerate transport kinetics in amine-functionalized systems, exceeding the productivity of reference commercial membranes by more than five times [18,31].

**Figure 4:**
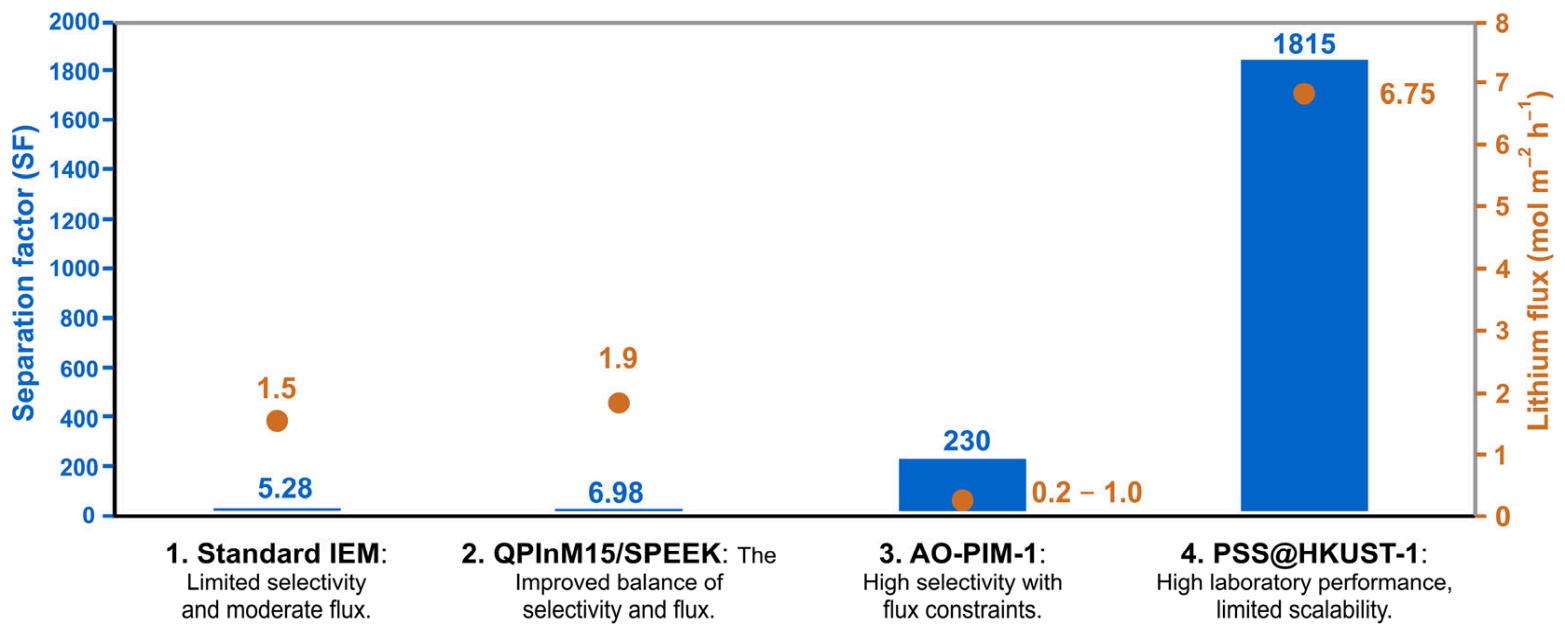
Comparative analysis of separation factor and lithium flux for representative membrane systems reported in this review. Each point represents a literature-reported system evaluated under the conditions specified in the source. The figure illustrates relative performance trends rather than defining a common quantitative scale or a universal ranking across membrane classes.

To contextualize the performance of the architectures discussed in this section, Figure 4 presents a benchmarking analysis that contrasts the separation factor (SF) against productivity (permeation flux) for the Li^+^/Mg^2+^ pair. This representation allows for the identification of the operational balance reached by quaternized Tröger’s base membranes (QPInM15/SPEEK), which achieve a favorable balance with a factor of 6.98 and the highest reported flux of 1.9 mol m^−2^ h^−1^. This performance shifts the conventional trade-off boundary, exceeding commercial ion-exchange membranes (IEM, SF≈5.28) and offering greater industrial viability than crystalline framework structures (MOFs/COFs). The latter, despite reaching high selectivities, exhibit low permeation fluxes for large-scale applications (e.g., 0.038 mol m^−2^ h^−1^ for TpBDMe2 structures).

**Table 3:** Separation factors and ionic flux across membrane systems from the five research streams under reported experimental conditions.

Membrane System	Target Cations	Driving Force/Operating Mode	Solution Matrix	Selectivity/Separation Factor (SF)	Flux/Permeability	Ref.
ZIOS (18 μm selective layer)	Cu^2+^ vs. Ni^2+^, Co^2+^, Mn^2+^	Passive Adsorption/Coordination Kinetics	Equimolar mixture, pH 2.5	Cu^2+^/Ni^2+^ ≈ 193; Cu^2+^/Mn^2+^ ≈ 2049; Cu^2+^/Co^2+^ ≈ 638	Highly selective adsorption efficiency	[31]
PSS@HKUST-1 (MOF)	Li^+^ vs. Mg^2+^	Active Electric Field (Electrodialysis)	Binary (Electric field)	Li^+^/Mg^2+^ = 1815	6.75 mol m^−2^ h^−1^	[37]
DMBP-QTB (Rigid PIM)	Li^+^, Na^+^ vs. Mg^2+^	Passive Diffusion (Diffusion Dialysis)	Binary mixtures	Na^+^/Mg^2+^ = 161.3; Li^+^/Mg^2+^ = 152.2	Cutoff size ≈ 8.2 Å	[66]
PFOAM50 (Trioctylamine)	Bi^3+^ vs. Cu^2+^	Electro-assisted Dialysis (PIM-ED)	HCl 0.3 M (Electrodialysis)	Bi^3+^/Cu^2+^ ≈ 471.4	131.0 × 10^−2^ mmol m^−2^ h^−1^	[18]
[MTOA^+^][Cl^−^]/PVC (PILIM)	Zn^2+^ vs. Cu^2+^, Fe^2+^	Passive Pertraction (Liquid Membrane)	HCl 1 M (Pertraction)	Zn^2+^/Cu^2+^ = 1996; Zn^2+^/Fe^2+^ = 606	Diffusion-controlled kinetics	[51]
QPInM15/SPEEK (Blending)	Li^+^ vs. Mg^2+^	Active Electric Field (Electrodialysis)	Binary (Electrodialysis)	Li^+^/Mg^2+^ = 6.98	1.9 mol m^−2^ h^−1^	[63]
CAIP-MOF (CMM)	Li^+^ vs. Mg^2+^	Pressure-driven Nanofiltration (NF)	MLR 10:1 (2000 ppm)	Li^+^, Mg^2+^ = 108	15.4 LMH/bar (Water)	[74]
CEM (Donnan Dialysis)	Li^+^ concentration	Concentration-driven Donnan Dialysis	Synthetic eluate (30 mM)	7.6–18.9× -fold (Conc. factor)	22–74% Recovery	[74]
TpBD-[(SO_3_H)_2_]0.67 (COF)	Mn^2+^, Co^2+^, Ni^2+^	Passive Diffusion (Diffusion Dialysis)	Ternary + EDTA (Dialysis)	Permeate ratios ≈ 8.17:7.14:1	27.26 mmol m^−2^ h^−1^	[75]
TpPa-AO (Bioinspired)	Cu^2+^ vs. Fe^3+^, Zn^2+^	Passive Adsorption/Coordination Kinetics	Equimolar ternary	Cu^2+^ > Fe^3+^, Zn^2+^ (Selective adsorption)		[3]

### 4.5. The Laboratory-to-Industry Gap

A common pattern is shared by the four streams described in Section 4.1, Section 4.2, Section 4.3 and Section 4.4, regardless of their mechanistic differences. The highest performances reported in the literature invariably correspond to controlled laboratory conditions: simple synthetic solutions, constant temperature, short operating periods, and membrane areas of a few square centimeters. The question of whether these performances are maintained, degraded, or collapse under real industrial operating conditions is the most pressing boundary condition in the field, and the available evidence remains more limited for industrial operation than for laboratory performance [1].

This gap is not merely quantitative. Under real industrial conditions, simultaneous heterogeneous perturbations are introduced. Multi-ionic competition involving dozens of species in solution, organic and inorganic fouling on the membrane surface, pH and temperature fluctuations, longitudinal concentration gradients in large-area modules, and accumulated chemical and mechanical degradation over months or years of operation are examples of such perturbations. None of these acts independently, and their combined effects on membrane selectivity and stability cannot be predicted by simple superposition of the individual effects studied in the laboratory [16].

Although high separation factors are important, they do not by themselves establish industrial relevance. Practical deployment also depends on membrane lifetime, recovery yield, productivity, energy consumption or current efficiency, effective membrane area, fouling resistance, and the composition and variability of the feed stream. Accordingly, the present framework evaluates selectivity as one component of a broader performance envelope rather than as a standalone indicator of industrial viability.

#### 4.5.1. Quantified Dimensions of the Gap

The difference between laboratory and industrial performance is manifested as a loss of structural integrity under continuous flow. Stable selectivities have been reported in micro-scale prototypes; however, in applications with real brines (409 g/L TDS), polymeric chains rearrange, increasing the intermolecular distance from 6.3 to 7.1 Å. This structural plasticity causes K^+^/Mg^2+^ selectivity to collapse from 16.60 to 5.67 after prolonged operating periods, showing that chemical durability is the limiting parameter for scalability [17].

The recent literature has documented the magnitude of performance degradation during the transition from ideal to real conditions. In PIM systems, Hernández-Fernández et al. [51] reported losses of up to 52% of the carrier weight after 85 days of continuous operation in 1 M HCl solution, a condition that, although aggressive, is representative of certain industrial hydrometallurgy effluents. Nowik-Zajc and Sabadash [8] compiled data from multiple PIM stability studies and found that operational lifespan under real conditions rarely exceeds three months, compared with the indefinite theoretical lifespan implied by steady-state transport models.

In IEM systems integrated into electrodialysis, the transition from laboratory cell to pilot module introduces non-uniform flow distribution effects and longitudinal concentration polarization that reduce effective selectivity between ions of identical charge by 20 to 40% compared to values obtained in a laboratory cell, according to data reported by Campione et al. [16] in pilot-scale electrodialysis systems. This degradation does not respond to changes in the membrane material but to scale hydrodynamic effects that laboratory designs do not reproduce, which are independent of the specific ion pair under separation.

For composite nanofiltration membranes applied to Li^+^ recovery from natural brines, Zhao et al. [44] noted that the presence of natural organic matter, frequently ignored in laboratory studies with synthetic solutions, generates surface fouling that alters the membrane’s effective charge and modifies selectivity in ways not predictable from laboratory data. Complementary evidence from systems designed specifically for real aqueous matrices shows that performance indicators derived from laboratory-grade synthetic solutions overestimate operational selectivity, with deviations that scale with the ionic complexity of the actual feed. This bias has a mechanistic basis: competing ions present in real matrices modify the local electrochemical environment at the membrane interface, altering both the partitioning coefficient and the diffusion selectivity in ways not captured by binary-solution calibration data [76]. In matrices as complex as seawater or salt-lake brines, Macías and Rodríguez de San Miguel [45] showed that separation factors for heavy metals in synthetic solutions are reduced by 30–70% in real matrices, depending on the membrane system and the specific brine composition.

**Figure 5:**
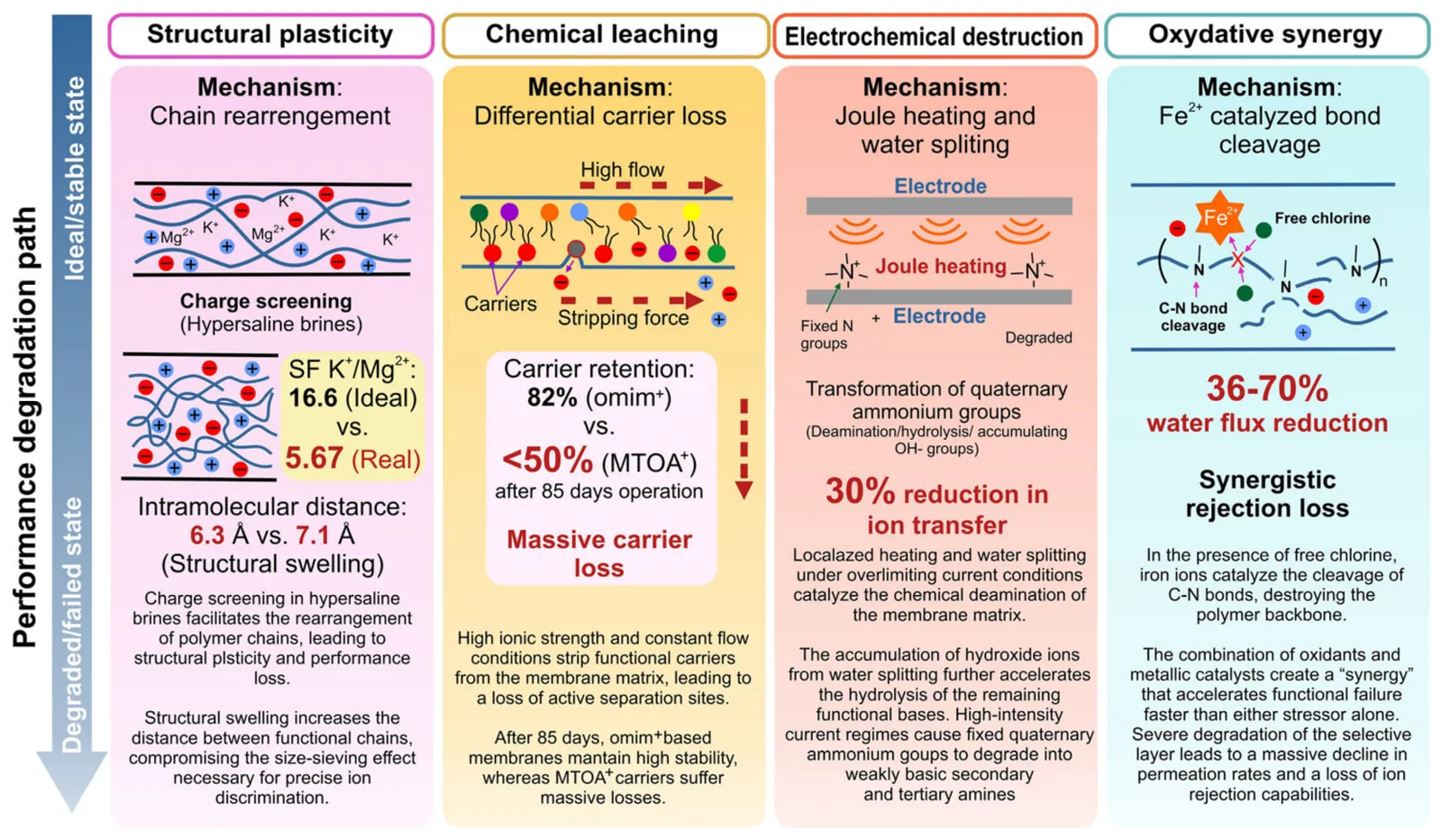
Failure-mode synthesis of representative degradation pathways in polymer-based selective membranes. The panels illustrate experimentally reported responses associated with structural plasticity, chemical leaching, electrochemical destruction, and oxidative synergy. These metrics are not directly comparable in magnitude and are presented here as distinct failure responses rather than as a single degradation trajectory [8,17,51,77].

Figure 5 summarizes representative failure modes reported for polymer-based selective membranes under realistic operating conditions. The figure is intended as a conceptual synthesis of distinct degradation pathways rather than as a unified quantitative trajectory, because each panel captures a different experimentally reported response metric [8,51].

#### 4.5.2. Determining Factors of the Gap

The magnitude of the laboratory-to-industry gap is determined by five factors in polymeric membranes for ion separation, ordered by their frequency of report as the primary cause of degradation: (a) the loss or degradation of the active selectivity element, such as the carrier in PIMs, functional groups in IEMs, or nanopore integrity in nanostructured membranes (this factor is the most documented and directly compromises selectivity) [8,51]; (b) surface fouling, which alters effective charge, blocks pores, and modifies transport kinetics progressively and irreversibly in the absence of adequate cleaning protocols [1]; (c) scale hydrodynamic effects, such as non-uniform flow distribution, dead zones, and longitudinal concentration gradients, which introduce operational heterogeneity absent in laboratory cells [16]; (d) real multi-ionic competition, whose intensity in industrial matrices exceeds laboratory test conditions, with a consequent effect on effective separation factors [40,45] and (e) accumulated mechanical degradation under cycles of pressure, temperature, and pH variations, which compromises the structural integrity of membranes designed for controlled static conditions [68].

It has been quantified that certain ionic liquids retain 82% of their weight after 85 days in acid media, whereas other systems lose more than 50%, compromising the technology’s economic viability [10,51]. Furthermore, the lack of standardized testing protocols generates inconsistencies; e.g., a membrane with 99.9% rejection at the laboratory scale can degrade when scaled to industrial modules due to the formation of micro-defects undetectable with current methods [37]. On the other hand, for framework-based architectures, scalability is additionally constrained by the high cost of organic building block synthesis, which currently remains incompatible with commodity-scale applications despite recent progress toward ton-scale COF production through mechanochemical and flux synthesis methods [78]. This advantage remains contingent on the operating regime, membrane stability, and feed complexity, so it should not be interpreted as a universal ranking across all membrane families.

#### 4.5.3. Reported Mitigation Strategies and Their Limitations

Several strategies have been reported to reduce the gap, although none eliminate it at the field’s current stage. Mechanistic hybridization, which combines electrical driving force with carrier chemical selectivity, can improve operational robustness by reducing dependence on concentration gradients that fluctuate in real conditions [18]. The interfacial functionalization of composite membranes improves mechanical stability and reduces the loss of the active component, according to Le et al. [31]. The design of modules with optimized geometry for uniform flow distribution reduces scale hydrodynamic effects [16]. However, none of these strategies have been validated in real industrial conditions for operating periods exceeding six months. The absence of long-term durability data in representative conditions is the most evident gap in the field as a whole. Consequently, the industrial adoption of high-selectivity ionic polymeric membranes advances more slowly than their laboratory development.

## 5. Integrative Discussion and Conceptual Framework

The same object of study is shared by the five research streams described in Section 4, ion selectivity in polymeric membranes, but they approach it from different mechanistic principles, architectural scales, and performance criteria. This diversity of approaches is a strength of the field in exploring the design space, but simultaneously poses an integration problem, since results reported from each stream are difficult to compare, theoretical divergences between them are not mapped, and accumulated knowledge has not yet crystallized into generalizable design principles. This section builds that integration at three progressive levels of abstraction. Section 5.1 cross-compares the five streams across common analytical dimensions, identifying patterns not visible from within any particular stream. Section 5.2 maps the sources of theoretical conflict that explain why the field produces results that are difficult to compare and why performance predictions have limited validity outside the conditions in which they were generated. Section 5.3 proposes a three-dimensional framework that emerges from these patterns and conflicts, offering common analytical dimensions to position heterogeneous systems in a shared design space, read their limitations in an integrated manner, and formulate testable predictions regarding which design combinations will be most effective under specific conditions.

### 5.1. Cross-Mechanism Comparison

Cross-mechanism comparison shows that steric sieving and Donnan exclusion are insufficient to discriminate between cations of identical charge in complex matrices [25]. Framework architectures (MOFs/COFs) outperform amorphous polymers through their ability to cooperatively coordinate window size and pore-wall chemistry [70]. While in conventional polyamide membranes selectivity is governed by the Stokes radius, in sub-nanometric UiO-66 channels, it has been demonstrated that tuning ionic affinity via functional groups, such as –OMe, allows for the manipulation of the ionic dehydration barrier, reaching K^+^/Mg^2+^ selectivities of up to 1567.8. This shows that intracanal chemical interaction is not merely a complement but the primary driver of selectivity by thermodynamically compensating for the energetic penalty of desolvation [70].

The evaluated data indicate that ion selectivity in polymeric membranes operates through multiple mechanisms correlated with the membrane’s architectural scale. When the dominant selectivity mechanism, the architectural scale of operation, the type of driving force, the demonstrated operational stability, and the maturity for scalability are simultaneously contrasted through the same analytical dimensions, a pattern is revealed that is not visible from within any particular stream (Table 4).

**Table 4:** Cross-comparison of the five research streams across mechanism, operational maturity, and design limitations.

	PIMs	IEM/Electrodialysis	Nanostructured Membranes	Composite Design	PIM-ED Hybridization
Dominant mechanism	Chemical recognition (carrier)	Electrostatic exclusion (Donnan)	Steric-hydration sieving	Multi-mechanistic by design	Facilitated transport + electromigration
Architectural scale	Molecular (carrier in matrix)	Mesoscopic (charge in polymer network)	Sub-nanometric (defined pore)	Variable (depends on component)	Molecular + mesoscopic
Driving force	Concentration/activity gradient	External electric field	Pressure or concentration gradient	Concentration or pressure gradient	Electric field + activity gradient
Representative selectivity performance	High selectivity under favorable carrier loading and target-ion matching [51]	Moderate selectivity for ions of identical charge	High selectivity under well-controlled pore geometry and feed composition [65]	Variable selectivity depending on the specific modification	Very high selectivity in selected laboratory systems [18]
Operational stability	Weak–moderate, limited by carrier loss [51,58]	Moderate–good under controlled current density [77]	Moderate, with residual heterogeneity [68]	Moderate; depending on interface quality	Emerging data; not yet consolidated
Scalability maturity	Low–medium	High (established industrial technology)	Low	Medium	Very low (laboratory stage)

Note: Representative selectivity performance is reported qualitatively to preserve comparability across different driving forces and operating conditions.

An apparent inverse relationship is observed between mechanism specificity and technological maturity in the systems reviewed. IEM-ED, which operates through electrostatic exclusion, is the most mature and scalable technology in the dataset. PIMs and nanostructured systems remain laboratory-scale systems because their performance is more sensitive to matrix complexity and long-term stability. This relationship reflects the manufacturing control requirements imposed by mechanism complexity, which scale poorly. The design of systems with both high specificity and high scalability remains unresolved in any individual stream.

Operational stability is a common limitation across all streams. Regardless of the mechanism, all streams show performance degradation under sustained real conditions. In PIMs, the issue is carrier loss [51,58]; in IEMs, the electrochemical destruction of functional groups at high current density [77]; and in nanostructured membranes, structural instability in complex matrices [68]. This convergence at the point of failure suggests that operational stability is a systemic limitation requiring transversal rather than incremental design strategies.

Hybrid systems are not merely the sum of their components. The PIM-ED case, though in early stages, illustrates that combining mechanisms can produce properties not exhibited by either component separately [18]. The high separation factors reported cannot be explained by the selectivity of the PIM alone or by electrodialysis alone. It results from the combined action of carrier chemical recognition and field-driven transport. This has a direct design implication because the architecture of the coupling between mechanisms, rather than just material selection alone, is a first-order design parameter.

### 5.2. Sources of Theoretical Conflict

The cross-comparison in Section 5.1 reveals that differences between streams are not simply a matter of material or application, but reflect deeper theoretical divergences on how selectivity is measured, modeled, and defined. From this analysis, three sources of theoretical divergence emerge:

1. Divergence by level of description. Ion transport models through membranes operate in two registers that are rarely articulated. Continuum models, modern formulations of the Nernst–Planck model extended with dielectric exclusion (DSPM-DE) and its variants, assume averaged membrane properties because of uniform fixed charge density, stationary pore size distribution, and constant diffusion coefficients. These formulations provide analytical tractability for predicting ionic rejection in nanofiltration and electrodialysis, though without capturing the specificity of ion-membrane interactions at the molecular level [40]. In contrast, molecular models, including Molecular Dynamics (MD) and Density Functional Theory (DFT), accurately capture specific hydration energies for each ion and free energy barriers for transport, but they correspond to idealized systems of perfectly defined nanopores, far from the real structural heterogeneity of polymeric membranes [25].

The field operates with two parallel languages that cite each other but do not functionally integrate. Experimental selectivity is reported in terms of macroscopic separation factors, while hydration energies and ionic transfer barriers at the atomic scale are reported in computational studies. Liu et al. [25] explicitly recognized this difficulty, noting that connecting computational predictions with the selectivity observed in real membranes remains an unresolved challenge. Until a multi-scale integrative framework exists, an active area, though still without consensus [7], this divergence will persist as a source of interpretative ambiguity.

2. Divergence by measurement conditions. Selectivity reported in synthetic binary solutions frequently differs from that observed in multi-ionic systems or real matrices. Macías and Rodríguez de San Miguel [45] demonstrated that the simultaneous presence of competing cations in seawater modifies the separation factors of Pb^2+^, Cd^2+^, and Zn^2+^. Occasionally, such effects invert the selectivity order between ions compared to values obtained in synthetic solutions with a single competitor. This result cannot be predicted by models built with binary data, which assume independence between individual ionic fluxes, a premise violated under high multi-ionic competition [40]. Studies specifically designed around water treatment matrices confirm this divergence quantitatively. The selectivity metrics obtained under controlled laboratory conditions fail to predict performance in real effluent streams even when the dominant ion pairs are nominally identical, because the multi-component background modulates the membrane’s effective charge state and transport geometry [76].

The mechanistic reason is that membrane structure under real operation differs qualitatively from its static structure. Tong et al. [15] demonstrated in piperazine nanofiltration membranes that operating conditions can expand the effective pore radius by up to 60% and increase volumetric charge density 4–5 times compared to the no-flow state. Wang et al. [13] confirmed, in hydrated microporous membranes, that the geometry of transport channels under hydration redefines design parameters compared to the dry state. Consequently, the data support treating selectivity as an operational state variable of the combined membrane–solution–operating conditions system. The implication for scientific reporting is that a separation factor without precise specification of operating conditions, feed concentration, pH, temperature, competing ions, pressure, or current density is data of limited comparability across the literature [8].

To systematically address these operational limitations, Table 5 compiles a failure-mode synthesis that categorizes the distinct, non-directly comparable physical, chemical, and electrochemical degradation mechanisms of polymer-based selective membranes under realistic operating conditions. Rather than assuming a unified or directly comparable kinetic degradation trajectory, which would introduce severe dimensional inconsistencies, this synthesis segregates failure modes by specific degradation descriptors across three major membrane classes (IEMs, PIMs, and Composites) [23,31,41].

3. Divergence by performance criterion. The third source of conflict is thermodynamic. In polymeric membranes for ion separation, the same structural factors that increase permeability (higher free volume, lower cross-linking density) tend to reduce discrimination between species of similar properties. DuChanois et al. [7] formalized this tension for ion separation membranes, identifying that conventional polymeric membranes are limited by the heterogeneity of their free volumes, and that overcoming this limitation requires either precise molecular control over pore distribution or the incorporation of specifically designed ion-membrane interactions.

Saif et al. [69] quantified this tension with experimental precision. In SPES/HMO composite membranes for Li^+^/Mg^2+^ separation, applying 0.2 V externally increased Li^+^ flux nearly fivefold but reduced the separation factor from 9.10 to 5.0. Electrical driving force accelerates the transport of both ions, but with unequal effects on relative selectivity, an expression of the more general principle that increasing active non-equilibrium reduces discrimination between species that do not radically differ in their transport properties. Wang et al. [18], in the PIM-ED system for Bi^3+^ separation, partially mitigated this trade-off through the combined action of carrier chemical recognition and the electric field, achieving simultaneously high flux and high permselectivity, a result indicating that mechanistic hybridization can, under specific conditions, shift the operational boundary beyond the individual limitations of each component.

The methodological consequence is that comparing the separation factor of a PIM under a passive concentration gradient with that of an IEM system under an intense electric field is equivalent to comparing different points on different performance curves, not equivalent materials. The absence of standardized reporting protocols that simultaneously include flux, selectivity, and complete operating conditions perpetuates comparative ambiguity in the field [7,8].

**Table 5:** Failure-mode synthesis: Classification of functional degradation mechanisms and quantitative metrics in polymer-based selective membranes compiled from the primary literature.

Membrane Class	Degradation Descriptor	Operating Condition	Main Failure Mode	Representative Quantitative Observation	Ref.
IEM	Structural plasticity	High ionic strength/real brine (Llullaillaco Salt Lake, Argentina, 409 g/L TDS)	Counter-ion shielding and chain rearrangement of the PEI active layer	Intermolecular distance (XRD) expansion from 6.3 Å to 7.1 Å due to ionic screening	[17]
IEM	Selectivity loss	Electrodialysis in hypersaline matrices	Loss of active surface layer charge density and deterioration of ionic crosslinking	K^+^/Mg^2+^ separation factor drops from 16.60 to 5.67 after 30 days of operation	[17]
PIM	Chemical leaching (Unstable carrier)	1 M HCl exposure (85 days/2048 h)	Dissolution of quaternary ammonium carrier due to poor compatibility with polymer matrix	[MTOA^+^][Cl^−^] carrier mass retention drops below 50% due to phase-separation and leaching	[51]
PIM	Chemical leaching (stable carrier)	1 M HCl exposure (85 days/2048 h)	Enhanced hydrophobic immobilization of imidazolio within PVC matrix	[omim^+^][PF_6_^−^] carrier retains 82% of its initial weight	[51]
Both	Functional group destruction	Overlimiting current density/localized Joule heating	Irreversible thermal and chemical deamination of strongly basic quaternary ammonium sites	Conversion of strongly basic sites to weak amines, increasing the share of inactive surface (α) from 4% to 17% in heterogeneous MA-41 membranes5	[74]
Composite	Interface delamination	Continuous electrodialysis for transition-metal or lithium separation	Blending stability decay and loss of interfacial adhesion between SPES active phase and polymer backbone	HMO-reinforced (20 wt.%) composite membranes suppress delamination, maintaining a stable lithium flux of 9.1 mol/(m^2^ h)	[8]
IEM	Interfacial Mineral Scaling	Donnan dialysis concentration using alkaline draw solutions (NaHCO_3_/Na_2_CO_3_ at pH 11.5)	Competitive transport of divalent counter-ions leading to precipitation of magnesium/calcium carbonates	Precipitation of mineral scaling on the draw side of the membrane, restricting mass transfer and halting monovalent	[60]

#### Convergence of the Three Divergences

The interactions between these three areas of divergence limit our ability to predict how selectivity changes under varying operating conditions (divergence 1 to 2). The dependence of selectivity on these conditions means the flux–selectivity trade-off can take different forms in each system and at each operating point (divergence 2 to 3). Moreover, the absence of standardized reporting protocols perpetuates ambiguity in cross-study comparisons, because available data are not in the format that allows calibration or systematic comparison (divergence 3 to 1). This feedback loop sustains the fragmented state of the field, and it is what the conceptual framework in Section 5.3 seeks to interrupt.

The current theoretical debate converges on three fundamental divergences that membrane design must address: (i) the scale divergence, where continuum models (e.g., DSPM-DE) fail to capture dielectric constant fluctuations in <1 nm pores, which can drop from 80 to values below 40, doubling rejection rates without altering geometry [7]; (ii) the matrix divergence, where successes achieved in laboratory binary solutions often do not translate directly to industrial conditions due to charge screening and multi-ion competition [2,79]; and (iii) the temporal divergence, driven by structural plasticity. Experimental data confirm that hydrodynamic forces expand the effective pore radius by up to 60% during operation, while bound water in the polymer acts as a dynamic mediator that can enhance diffusion selectivity under certain charge-density regimes [30].

### 5.3. Proposed Conceptual Framework

The synthesis of the five themes in Section 4 and the analysis of the theoretical divergences in Section 5.2 support the proposal of a framework that primarily organizes rather than being strictly predictive. It offers common analytical dimensions to compare membrane systems operating under different principles, identify their limitations, and guide design decisions toward combinations with a higher probability of success under real operating conditions. The framework is expressed through three partially coupled dimensions that together define the design space of ion-selective polymeric membranes:

Dimension 1: Spectrum of selectivity mechanisms. This dimension describes the type of mechanism governing ionic discrimination, organized as a continuous spectrum with two poles. At the electrostatic pole, selectivity is dominated by ion charge and is governed primarily by the membrane’s Donnan potential (discrimination between cations and anions) but without the capacity to differentiate between ions of identical charge. At the molecular recognition pole, selectivity depends on specific ion–carrier or ion–pore interactions sensitive to coordination geometry, desolvation energy, and the specific chemical affinity of the ion–active site pair, enabling discrimination between ions of identical charge and similar physicochemical properties.

The five streams described in Section 4 are positioned at different points of this spectrum. Conventional IEMs operate near the electrostatic pole; PIMs with high-specificity carriers operate near the molecular recognition pole; nanostructured membranes and composite systems occupy intermediate positions depending on the specific design [7,19]. This dimension suggests that the ability to discriminate between ions of similar properties, such as Li^+^/Na^+^, Li^+^/Mg^2+^, or Cu^2+^/Zn^2+^, increases as one moves toward the molecular recognition pole, but at the cost of higher design complexity, lower operational robustness, and lower technological maturity. For the selective recovery of Lithium from brines with high Na^+^ and Mg^2+^ concentrations, this dimension establishes that systems positioned toward the molecular recognition pole are mechanistically the most suitable candidates, though they are also the most distant from immediate industrial application.

Dimension 2: Dynamic state of the membrane. The second dimension describes the degree to which membrane structure and selectivity change under real operating conditions. At the static pole, the membrane behaves approximately as an invariant filter whose transport properties are largely constant under a defined reference condition, but not fully independent of environmental effects. At the responsive pole, the membrane structure dynamically adapts to operating conditions, modifying its selectivity in ways that can be favorable or unfavorable according to the design [74].

The experimental data compiled in Section 4 and Section 5.2 indicate operational deviations from the static pole. Wang et al. [13] demonstrated that pore hydration redefines transport geometry compared to the dry state; Tong et al. [15] quantified effective pore radius expansion under operating pressure; Espinoza et al. [30] showed that bound water in highly charged membranes acts as a dynamic mediator of selectivity. The relevant design question is not whether the membrane is dynamic, but whether this structural dynamics is under the designer’s control or constitutes an uncontrolled source of performance variability [80].

Controlled structural plasticity is an active frontier for overcoming the limitations of static filtration. One advancement involves bioinspired membranes functionalized with dual-responsive microgels acting as intelligent water gates. These systems demonstrate that transport channels can be actively regulated through thermo- and ion-recognition triggers, achieving distribution coefficients (Kd) near 1000 mL/g for the selective interception of Pb^2+^ ions. Such architectures validate the conceptualization of selectivity as a dynamic state, where the membrane structure evolves in response to the chemical environment to enhance selectivity [67].

This dimension introduces a distinction with direct implications for experimental design. Characterizing a membrane solely under static or low hydraulic load conditions and reporting those values as representative performance metrics is a practice that overestimates real selectivity under operating conditions and contributes to the laboratory-to-industry gap examined in Section 4.5.1. Rational design must incorporate characterization of the membrane’s dynamic state under target process operating conditions [8,16].

Dimension 3: System complexity. This dimension describes the complexity of the separation matrix for which the membrane system has been designed and characterized. At the low-complexity pole, the system is characterized by synthetic binary solutions of a single ion pair at controlled concentrations and standard laboratory conditions. At the high-complexity pole, the system is characterized by real industrial matrices containing multiple competing ions, organic matter, pH and temperature variations, and unpredictable feed-composition perturbations.

A system’s position in this dimension not only describes separation difficulty but also conditions whereby mechanisms from Dimensions 1 and 2 remain operative and are attenuated. Macías and Rodríguez de San Miguel [45] demonstrated that matrix complexity reduces separation factors by 30 to 70% compared to synthetic solution values; Ozkul et al. [40] established that dominant transport mechanisms in ED change qualitatively as feed ionic complexity increases.

**Figure 6:**
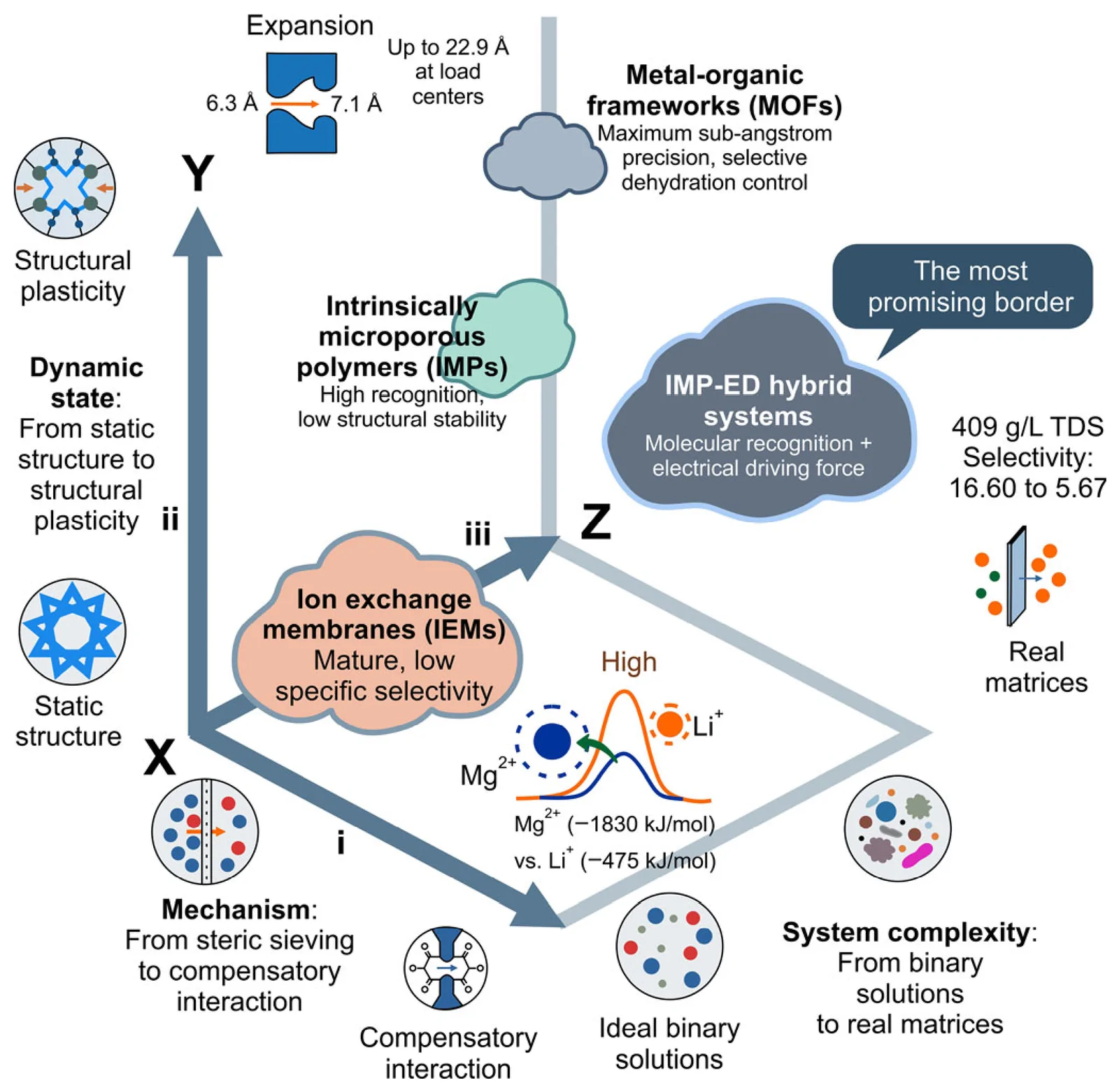
Three-dimensional analytical framework for ion-selective polymeric membranes. The three axes represent (i) mechanism, (ii) dynamic state, and (iii) system complexity. The framework positions membrane systems within a unified design space that links transport mechanism, structural response under operating conditions, and feed-matrix complexity.

The convergence of the five research streams analyzed in Section 5 into a unified design language requires a representational framework in which the three axes along which ionic selectivity varies as a system-level property are made explicit. In Figure 6, the three-dimensional analytical framework proposed in this review is presented. The three axes of the design volume are assigned to the three critical dimensions identified throughout the preceding analysis: Dimension 1, mechanism, is associated with the transition from global electrostatic exclusion toward compensatory chemical interaction as the dominant transport-controlling principle; Dimension 2, dynamic state, is associated with the structural response of the membrane architecture to operating stress, from rigid crystalline frameworks to plasticity-prone polymeric matrices under hypersaline flow; and Dimension 3, system complexity, is associated with the chemical environment of the separation, from idealized binary solutions to real multicomponent industrial matrices. By locating a given membrane system within this three-dimensional space, its mechanistic basis, structural stability under real conditions, and performance transferability to industrial applications can be identified simultaneously.

#### 5.3.1. Positioning of the Five Streams

This three-dimensional framework is presented as a semi-quantitative comparative map rather than an orthogonal design space. Its axes are useful for organizing the literature, but they remain partially coupled in practice because operating conditions influence both membrane dynamics and apparent selectivity. Table 6 synthesizes the positioning of the five streams reviewed across the three dimensions, using a three-level qualitative scale (low/medium/high) as a semi-quantitative coding scheme for each dimensional pole. Low, medium, and high denote semi-quantitative operational descriptors derived from the reviewed literature and are intended for conceptual positioning rather than universal classification.

The table shows that no individual stream simultaneously occupies favorable positions in all three dimensions. IEM/ED has high validated complexity but low molecular recognition power. PIMs have high molecular recognition but low dynamic stability and low validation in real matrices. Nanostructured membranes have the greatest mechanistic potential but the lowest operational maturity. PIM-ED systems are the most promising direction because their design seeks to combine the molecular recognition of PIMs with the operational stability that the electric field can provide. However, their validation under high-complexity conditions remains incipient [18].

Under the proposed three-dimensional framework, research streams occupy a trade-off spectrum between precision and stability. Crystalline framework membranes (MOFs/COFs) occupy the apex of the Mechanism dimension due to their sub-ångström control, but face challenges in the complexity dimension due to hydrolytic vulnerability [8,14]. Hybrid PIM-ED systems stand as the most promising direction for industry, combining high electrical productivity with bismuth fluxes (see Section 4.2.2) and shifting the Robeson upper bound through the fixed-site jumping mechanism [18,37].

#### 5.3.2. Framework Predictions

The three-dimensional framework suggests three testable hypotheses that can be experimentally evaluated:

**Hypothesis** **1.** *Systems that combine molecular recognition mechanisms (Dimension 1, high pole) with architectures that actively control their dynamic state (Dimension 2, controlled responsive pole) are expected to show higher and more stable Li^+^/Mg^2+^ selectivities than systems optimizing only one of these dimensions. This prediction is testable through the systematic comparison of conventional PIMs, cross-linked PIMs, and PIM-ED systems under identical solution and temperature conditions.*

**Hypothesis** **2.** *Selectivity degradation upon increasing system complexity (Dimension 3) may be greater under comparable conditions for systems positioned at the molecular recognition pole (Dimension 1) than for those at the electrostatic pole, because chemical specificity is more sensitive to ionic competition than electrostatic exclusion. This prediction is testable through selectivity experiments across a series of increasingly complex matrices using the same membrane systems.*

**Hypothesis** **3.** *Characterizing the membrane’s dynamic state (Dimension 2), under target process operating conditions, may be a more reliable predictor of real performance than membrane properties in a reference state. This prediction is testable by comparing selectivity data obtained from the same systems under static characterization and representative operating conditions.*

These three hypotheses emerge from the theoretical tensions identified in Section 5.2 and the patterns observed in the cross-comparison of Section 5.1. Their experimental verification is addressed in the research agenda developed in Section 6. Future work may explore bioinspired ligands and covalent carrier anchoring as strategies to improve transport energetics and mitigate leaching under realistic brine conditions [14,68].

To complement the three-dimensional framework, Table 7 summarizes representative cross-study dependencies linking operating variables to flux and selectivity across advanced polymeric membrane systems. Rather than implying direct numerical comparability, the table highlights recurring mechanistic trends under salinity, pressure, current density, pH, hydration state, membrane thickness, and exposure time, thereby providing a compact synthesis of the parameter sensitivity discussed throughout Section 3, Section 4 and Section 5.

## 6. Future Research Agenda

The theoretical discrepancies identified in Section 5 and the hypotheses formulated in Section 5.3 define a research agenda centered on identifying specific mechanistic behaviors that will determine the rational design of the next generation of selective separation systems. The five questions below are organized from operational challenges to mechanistic uncertainty, reflecting both the most immediate barriers to translation and the deeper questions that remain unresolved in the current literature. These are documented knowledge gaps rather than programmatic desires.

Question 1. How is the selectivity of polymeric membranes predicted and controlled in the dynamic operating state, rather than the static reference state? This question represents a primary operational challenge for research programs aiming to close the laboratory-to-industry gap. The evidence reviewed in Section 4.3 and Section 5.2 establishes that the structure of the membrane under operating conditions differs qualitatively from its structure in a dry or static state. Wang et al. [13] demonstrated that pore hydration redefines transport channels, Tong et al. [15] quantified pore radius expansions of up to 60% under operating pressure, and Espinoza et al. [30] established that bound water in charged membranes acts as a dynamic mediator of selectivity. No predictive framework yet allows calculation of operational selectivity from the structural properties of the membrane in a reference state. Addressing this question requires in situ characterization protocols under operating conditions, such as X-ray diffractometry in flow or time-resolved impedance spectroscopy, combined with transport models that incorporate the structural dynamics of the membrane as a state variable rather than a fixed parameter [7,25].

Question 2. What are the real limits of Li^+^/Mg^2+^ selectivity in polymeric membranes under representative brine matrices, and what membrane architecture approaches them most closely? Li^+^/Mg^2+^ separation presents technical constraints in lithium recovery from natural brines, as both ions are cations of different charges but with similar bare ionic radii (Li^+^: 0.76 Å; Mg^2+^: 0.72 Å) and very different hydrated radii (Li^+^: approximately 3.82 Å; Mg^2+^: approximately 4.28 Å). This makes hydration-based sieving the most promising mechanism but also the most sensitive to pore hydration conditions [13].

Li^+^/Mg^2+^ selectivity values reported in the literature vary by more than two orders of magnitude, from separation factors below 5 in conventional IEMs to values above 1000 in nanostructured membranes with controlled hydrophobic subnanometric pores [13,69], but most correspond to binary synthetic solutions. The question of how much of that selectivity is preserved in real brine matrices, with concentrations of Na^+^, K^+^, Ca^2+^, and organic matter characteristic of spent lithium battery effluents, remains unanswered in the literature [1,45].

Responding to this question requires an experimental matrix that combines at least three membrane families (modified IEM, PIM with Li^+^-specific carrier, and nanostructured membrane) evaluated under a series of matrices of increasing complexity, as per Prediction 2 of the framework in Section 5.3.2, using standardized reporting protocols that allow direct comparison between systems [8].

Question 3. Is it possible to design covalently anchored carriers in PIMs that maintain the complexation kinetics necessary for facilitated transport? Progressive carrier loss is one of the most reported limitations of PIMs and a major barrier to industrial adoption. Hernández-Fernández et al. [51] quantified losses of up to 52% of the carrier after 85 days of operation, and Nowik-Zając and Sabadash [8] established that a published PIM system has demonstrated operational stability exceeding three months under representative industrial conditions. Covalently anchoring the carrier raises a mechanistic trade-off, because facilitated transport requires both chemical recognition and sufficient carrier mobility.

The specific question is whether a design window exists in which the anchored carrier maintains sufficient local mobility for complexation and decomplexation kinetics without macroscopically diffusing into the aqueous phases. Recent work in supramolecular chemistry and in polymers with covalent chelating groups suggests that this window exists for certain ion–carrier pairs, but selective transport data in membranes with these systems are still scarce. Resolving this question would represent a major design advancement for PIMs, as it would decouple the chemical specificity of the carrier from its progressive loss into the aqueous phase, the most critical limitation of these membranes [8,83].

Question 4. Under what conditions does mechanistic hybridization (PIM-ED) consistently outperform the individual limitations of each component? Results from Wang et al. [18] on PIM-ED systems for Bi^3+^ separation suggest selectivity properties that are not observed in the corresponding individual components under the same conditions. However, those results correspond to laboratory conditions with synthetic solutions, and the mechanisms responsible for the observed performance gain have not been fully elucidated. The question is not whether hybridization works under ideal conditions, but under what operating conditions the hybridization advantage is maintained, when it degrades, and what coupling design parameters, such as carrier density, current density, base membrane composition, and feed composition, control that behavior [40].

Question 5. How is the debate over the carrier transport mechanism in PIMs resolved? This is one of the most fundamental from a mechanistic standpoint, although the least urgent for immediate application. The ambiguity between the two competing models, a mobile carrier that diffuses freely vs. ion–carrier complexes that jump between stationary sites, has direct implications for design. If the mobile carrier model is correct, the diffusivity of the carrier in the matrix would be the critical control parameter and would need to be optimized through plasticizer selection and cross-linking density. If the jumping model is more appropriate, the density and spatial distribution of the carrier sites would become the key parameters [24].

Macroscopic flux data do not discriminate between the two models because both predict similar dependencies of flux on concentration and activity gradients. Resolution requires characterization techniques that access carrier dynamics at the molecular scale under operating conditions. Examples include fluorescence correlation spectroscopy (FCS) to measure in situ carrier diffusivity, spatially resolved impedance spectroscopy to probe active site distribution, or molecular dynamics simulations calibrated with experimental transport data in model systems [25].

Together, these questions define a staged research program in which improved characterization, standardized reporting, and mechanistically informed design are the main prerequisites for closing the laboratory-to-industry gap.

### 6.1. Transition Toward Green and Circular Chemistry

Sustainability in membrane manufacturing should be treated as an important design parameter. Current investigations have evaluated the substitution of toxic solvents like NMP or DMF in favor of green alternatives such as Cyrene, ethanol, or ethyl acetate, and explored the use of biodegradable polymers like polylactic acid (PLA) or chitosan in PIM matrices [8]. Life cycle analysis (LCA) and techno-economic assessment (TEA) should be integrated from early development stages to help ensure that the performance advantages of MOFs and COFs are not eclipsed by prohibitive synthesis costs or long-term hydrolytic instability [84].

This transition may be supported by the integration of biopolymer-based hybrid nanomaterials. Recent studies on the interaction between chitosan-derived matrices and biomimetic membranes reveal that these bio-based materials provide a high density of functional sites for heavy metal coordination without compromising the integrity of the transport channels. This evidence suggests the feasibility of replacing toxic precursor solvents with environmentally compatible polymeric networks, ensuring that recovery processes align with the principles of circular chemistry [85].

Furthermore, the green transition requires addressing the inherent ecotoxicity and leaching of conventional PIM components. For example, although Aliquat 336 is widely used as a plasticizer, its corrosive and hazardous nature remains a concern [8]. To mitigate carrier leaching and toxicity, future studies must explore reactive ionic liquids with lower toxicity profiles, such as octylimidazole derivatives [50], or covalently tethered extractants [1]. Similarly, the substitution of toxic plasticizers like NPOE and DNOP is critical [53]. Recent evaluations showed that butyl stearate (BTS) represents a non-toxic, highly efficient alternative to NPOE in PVC and CTA formulations, improving heavy metal transport [53]. Furthermore, sustainable PIM fabrication must integrate biodegradable and renewable matrices [8], green plasticizers such as citrate esters [49], and bio-based solvents like ethyl acetate, 2-methyltetrahydrofuran, or Cyrene to ensure that the entire material lifecycle aligns with circular chemistry principles [49,57].

### 6.2. Scalability and Industrialization

Addressing the laboratory-to-industry gap requires standardized ion selectivity measurement protocols that include real multicomponent matrices rather than only ideal binary solutions [14]. Scalability is more plausibly supported by continuous manufacturing techniques, such as roll-to-roll processing for solution-processable PIMs or chemical vapor deposition (CVD) for defect-free 2D layers [33]. Rigorous validation of operational stability under high current density and prolonged convective flow is also needed, documenting carrier leaching mechanisms and structural collapse due to charge screening [29].

### 6.3. The Digital Separation Paradigm: Artificial Intelligence and Multi-Scale Modeling

The complexity of selectivity as a dynamic phenomenon has led to the integration of predictive frameworks based on artificial intelligence (AI) and machine learning (ML) to supplement empirical testing. Future work must prioritize multi-scale models that couple ab initio molecular dynamics (AIMD) simulations with continuum transport models, such as DSPM-DE, to capture real-time functional group protonation and environment-induced pore expansion. Interpretability analyses, such as SHAP values, must be implemented to identify which descriptors, including charge density, bound water, or dehydration energy, govern selectivity in specific sub-nanometric channels, enabling navigation through high-dimensional chemical spaces.

#### 6.3.1. Performance Prediction via Machine Learning (ML)

The field is increasingly moving from observation toward quantitative prediction through machine learning (ML). The recent literature shows that XGBoost-based models can predict SF for the Li^+^/Mg^2+^ pair with R^2^ = 0.88, using both intrinsic membrane descriptors, such as MWCO and surface charge, and operational descriptors, including flux and pressure [29,36]. This approach can identify non-linear patterns that traditional continuum transport models may fail to capture. Explainable artificial intelligence (XAI) frameworks, including SHAP (SHapley additive exPlanations) analysis, have suggested that ML can help identify the relative importance of size exclusion and electrostatic interactions in regulating ionic discrimination [86].

#### 6.3.2. Inverse Design and Physics-Informed Neural Networks (PINNs)

Inverse design modifies the screening protocol by defining permeability and selectivity targets before synthesis, allowing generative models, such as variational autoencoders, to propose candidate chemical architectures [59]. A key advancement is the integration of PINNs, which incorporate Nernst-Planck and Poisson equations into their loss function, helping digital design remain consistent with mass and charge conservation principles [37]. This may allow hybrid frameworks to explore the possibility of very high SF values for strategic metals by optimizing the location of specific coordination sites informed by DFT calculations [87]. PINNs have also shown potential for providing mechanistic interpretability for adaptive transitions between separation and fouling regimes, with the potential to outperform traditional semi-empirical models in some settings [46].

#### 6.3.3. Data Democratization: The Role of the Open Membrane Database (OMD)

The performance of AI in ion separation depends on the availability of high-quality data. The open membrane database (OMD) has become an important resource for global collaboration, providing curated NF and RO membrane datasets that comply with FAIR principles: findable, accessible, interoperable, and reusable [29]. Integrating these repositories with graph neural networks (GNNs) may help map the relationship between polymer backbone rigidity and ionic mobility, with the potential to reduce the trial-and-error approach that has long dominated the discipline [37].

**Figure 7:**
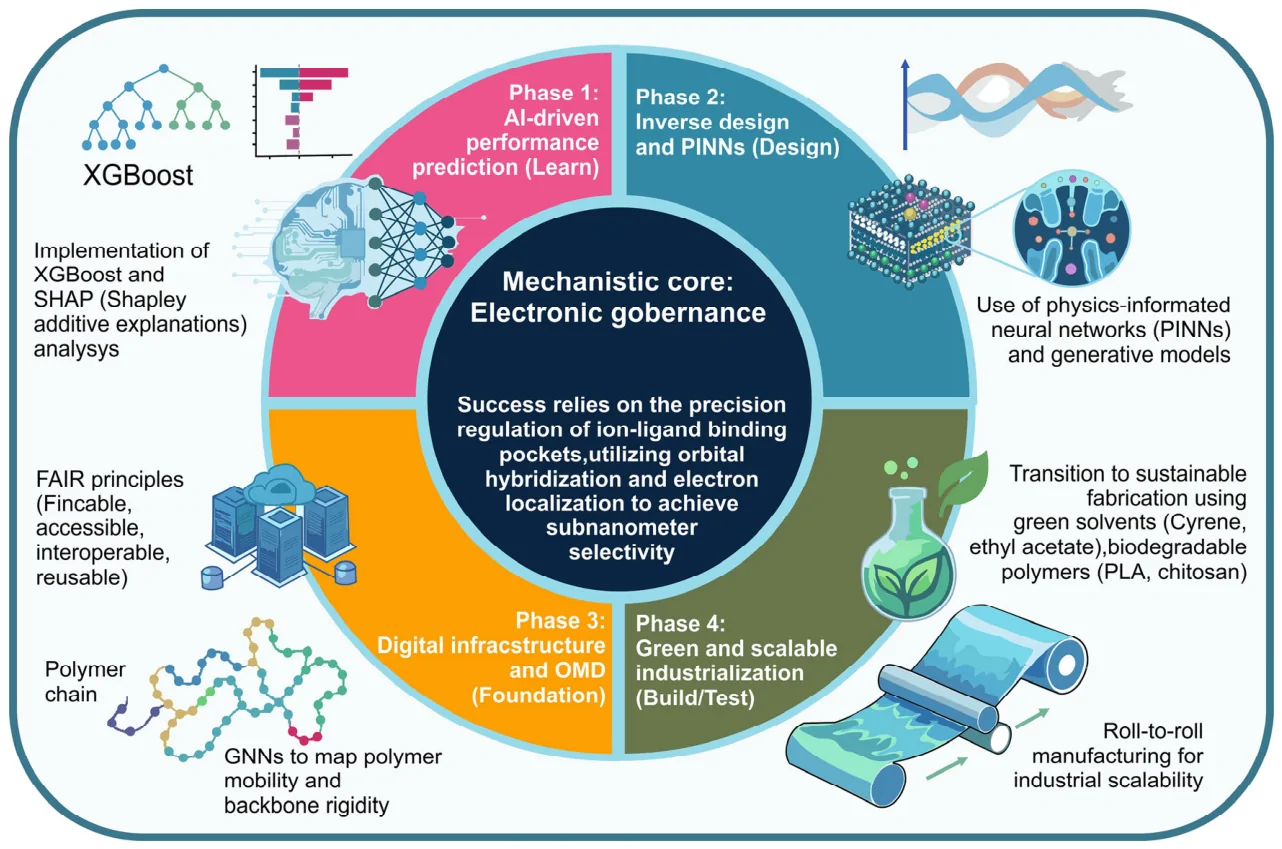
Research roadmap for the next generation of ion-selective membranes. The diagram integrates the future research agenda with the digital separation paradigm. (1) AI-driven performance prediction. XGBoost models and SHAP analysis predict Li^+^/Mg^2+^ separation factors with R^2^ = 0.88. (2) Inverse design and PINNs. Generative models and PINNs embed transport equations to maintain consistency in flux and selectivity profiles. (3) Digital infrastructure and OMD. The open membrane database, governed by FAIR principles, centralizes curated synthesis and performance datasets. (4) Green and scalable industrialization. Biopolymers, green solvents, and continuous processing support sustainable manufacturing to close the lab-to-industry gap.

Advancing beyond current limits requires an integrated roadmap that connects predictive modeling, inverse design, open-data infrastructure, and scalable manufacturing [27,30]. Figure 7 summarizes this transition from empirical development toward a digital separation paradigm grounded in machine learning, physics-informed modeling, and sustainable process design [29,37,49].

#### 6.3.4. Standardization and Data Scarcity in AI-Driven Membrane Inverse Design

Although ML and PINNs show promising potential in membrane inverse design, the transition toward the digital separation era is bottlenecked by a data infrastructure deficit. The membrane science community still lacks sufficiently standardized, open-access databases comparable to those established in other materials science domains. The existing literature is fragmented, with experimental performance metrics predominantly derived from idealized binary solutions tested under divergent hydrodynamic conditions. This lack of uniform metadata can limit the training and generalization capabilities of predictive algorithms. Models trained on heterogeneous literature may suffer from computational bias and often struggle to predict structural plasticity or selectivity collapse in real hypersaline brines [9]. The field must establish collaborative, open-source repositories governed by standardized reporting protocols for both synthesis parameters and multi-component operational testing [86].

## 7. Conclusions

A three-dimensional analytical framework has been proposed to organize ion-selective polymeric membrane systems according to transport mechanism, dynamic structural state, and separation-matrix complexity. Across the five research streams reviewed, selectivity was shown to arise from the coupled action of electrostatic exclusion, steric-hydration effects, and specific chemical recognition, with the relative contribution of each mechanism depending on membrane architecture and operating conditions. It was therefore established that membrane performance cannot be fully interpreted from dry-state characterization or from laboratory tests under simplified feeds, because structural plasticity, hydration state, and matrix complexity alter the transport landscape under process-relevant conditions.

The framework also makes it possible to position each membrane family within a common design space, showing that no single stream simultaneously occupies the most favorable region of all three dimensions. Conventional ion-exchange membranes remain industrially mature but mechanistically limited for same-charge discrimination, whereas polymer inclusion membranes and related carrier-mediated systems provide strong molecular recognition at the cost of lower operational robustness. Nanostructured and subnanometric membranes offer the highest mechanistic precision, although their transferability to complex matrices remains constrained by fabrication and stability issues. Composite and hybrid architectures, including PIM-ED systems, are therefore identified as the most promising route for bridging these limitations.

## Figures and Tables

**Table 1:** Comparative analysis of major previous reviews on selective ion separation membranes.

Review Reference	Covered Membrane Families and Core Scope	Key Transport Principles and Mechanisms Analyzed	Identified Gaps and Operational Limitations
Luo et al. (2018) [19]	Traditional ion-exchange membranes used in electrodialysis and electromembrane systems.	Donnan electrostatic exclusion, membrane potentials, and physical surface modifications.	Restricted strictly to electro-driven operations, omitting carrier-mediated transport in PIMs and precise sub-nanometer framework channels.
Tang and Bruening (2020) [1]	General ion separation mechanisms in pressure-driven, electro-driven, and concentration-driven systems.	Solute-tailored size sieving, electrostatic Donnan exclusion, concentration polarization, and uphill transport.	Primarily qualitative and phenomenological, lacking systematic multi-scale analysis of operational membrane degradation, swelling, and digital inverse design methodologies.
Stenina et al. (2020) [20]	Selectivity of transport processes in ion-exchange membranes, focusing on morphology-property links.	Ion exchange capacity, water uptake, polymer grafting, and layer-by-layer assembly for monovalent selectivity.	Strictly limited to conventional IEMs and electromembranes, ignoring emerging computational paradigms and carrier-mediated extraction thermodynamics.
Epsztein et al. (2020) [21]	Synthetic membranes with sub-nanometer pores compared with biological ion channels.	Dehydration-induced energy barriers, ion-pore interactions, and steric-electrostatic synergy.	Translating single-pore biomimetic transport mechanisms into scalable, mechanically stable macro-membranes remains a major bottleneck.
DuChanois et al. (2021) [7]	Advanced materials at the water-energy nexus, including polymers, 2D sheets, and biomimetic channels.	Transition-state theory, specific coordination chemistry (ligand binding), and free volume elements designed to bypass Robeson bounds.	High-level materials-focused approach, failing to systematically address dynamic membrane vulnerabilities and structural plasticization under real hypersaline stress.
Siekierka et al. (2021) [22]	Polymeric membrane modification strategies to enhance specific ion separation mechanisms.	Surface grafting, plasma treatment, blending, and chemical functionalization for targeted selectivity.	Qualitative and short review, lacking quantitative benchmarking of separation factors and a digital framework for performance prediction.
Fan et al. (2022) [23]	Emerging advanced materials designed to transition traditional IEMs to species-selective systems.	Electrostatic interaction, size sieving, charge density optimization, and surface barrier customization.	Limited strictly to conventional electromembrane processes, failing to compare transport thermodynamics with pressure-driven nanofiltration or carrier-mediated extraction.
Wang et al. (2023) [24]	Recent advances in polymer inclusion membranes for selective metal ion recovery.	Facilitated coupled transport, carrier–analyte complexation, and transition metal extraction configurations.	Limited strictly to carrier-mediated liquid or inclusion matrices, completely omitting comparison with pressure-driven nanofiltration or ordered crystalline channels.
Liu et al. (2024) [25]	Ion–ion selectivity in synthetic membranes featuring confined sub-nanometer structures.	Quantum confinement, dehydration energy barriers, surface charge density gating, and transport through 2D slits.	Excludes flexible polymeric backbones, organic–inorganic composite interfaces, and process-level industrial scalability or cost assessments.
Lou et al. (2025) [26]	State-of-the-art Covalent Organic Framework membranes for liquid-phase ion separation.	Physical confinement (size exclusion), electrostatic forces (Donnan), and chemical affinity (functional groups).	Highlighted solvothermal and interfacial fabrication barriers, alongside disordered interlamellar stacking that reduces mass transfer efficiency.
Cheng et al. (2025) [27]	Pore engineering in advanced crystalline frameworks (COFs and MOFs).	Building block symmetry, post-synthetic modification, localized charge density, and host–guest chelating or crown ether coordination.	Scalability limitations due to high precursor costs, weak interfacial adhesion, and the lack of quantitative structure-performance relationship models.
Ershov et al. (2025) [28]	Dense polymer and composite membranes analyzed under sub-nanometric water confinement.	Static and dynamic hydration properties, activation energy barrier scaling, and dehydration-promoted ion partitioning.	Lacks direct in situ experimental validation of real-time hydration states inside flexible, disordered industrial polymeric structures.
Yong et al. (2025) [29]	Nanofiltration membranes for efficient lithium extraction from complex salt-lake brines.	Ion dehydration, steric hindrance, electrostatic interactions, and process-level nanofiltration transport models.	Limited strictly to pressure-driven lithium–magnesium separations, omitting electromembrane processes and carrier-mediated extraction.
Ignacz et al. (2025) [9]	Data-driven technologies and Machine Learning for the advancement of membrane science since the 2000s.	High-throughput screening, direct and inverse generative design, and open databases such as the Open Membrane Database.	Heavy computational focus, lacking deep physical integration of thermodynamic ion dehydration and dynamic membrane degradation under real matrices.
You et al. (2026) [14]	Nanochannel membranes utilized for highly selective electrodialysis separations.	Confined transport within 1D and 2D sub-nanometer channels, zwitterionic gating, and electrostatic repulsion.	Focuses on idealized nanochannel architectures, leaving out practical industrial matrix effects and carrier-based transport systems.
Ben-Elijah et al. (2026) [2]	Membrane-based separations specifically designed for Rare Earth Elements recovery.	Supported liquid membranes, polymer inclusion membranes, and crystalline metal–organic frameworks.	Restricted strictly to lanthanide coordination chemistry, failing to establish a generalized cross-stream framework comparing REEs to other critical energy metals.

**Table 6:** Semi-quantitative descriptors used to position membrane systems within the proposed three-dimensional framework. Low, medium, and high represent heuristic categories derived from the literature and are intended for comparative organization rather than universal classification.

Classification Level	Dimension 1 (Transport Mechanism and Energetic Rules)	Dimension 2 (Dynamic Structural State and Swelling)	Dimension 3 (System Complexity and Saline Matrix)
Low	Donnan electrostatic exclusion without mandatory ion dehydration, characterized by a Debye length greater than or equal to the pore diameter [39,68,81].	Rigid or static crystalline structure with minimal pore variation under hydraulic pressure [32,60].	Dilute single-salt synthetic solutions under standard laboratory conditions [39,82].
Medium	Steric and hydration sieving, where the pore diameter is close to the hydrated ion size [21,23,81].	Controlled responsive behavior with reversible pore-diameter fluctuation under external stimuli [32,67].	Synthetic multicomponent matrices with moderate ionic strength [75,82].
High	Specific coordination at active sites within pores smaller than 1.0 nm, requiring severe dehydration compensated by chelating ligands [23,31,81].	Uncontrolled structural plasticity with pronounced swelling or active-carrier loss [47,51,63].	Complex hypersaline industrial effluents with strong competitive effects and foulants [60,63,82].

Note: The table indicates that no single stream occupies a favorable position in all three dimensions simultaneously.

**Table 7:** Cross-study sensitivity and operational dependencies of flux and species selectivity across advanced polymeric membranes.

Variable/Parameter	Reported Trend and Physicochemical Mechanism	Effect on Solute/Water Flux	Effect on Species Selectivity (SF)	Membrane System	Ref.
Salinity/Ionic Strength	Severe electrostatic shielding of membrane fixed charges by high ionic strength disrupts ionic cross-linking interactions between the surface-active layer and the underlying support matrix.	Increases membrane swelling and water uptake by a maximum value of 25%.	Substantial decline of monovalent/divalent cation selectivity (e.g., K^+^/Mg^2+^ SF drops from 16.60 to 5.67) under hypersaline conditions (409 g/L TDS real brine).	Selemion™ CSO (PEI/PS-DVB)	[17]
Applied Pressure	High transmembrane hydraulic pressure (2 to 8 bar) intensifies convective solvent transport through semi-flexible polymer networks, highlighting the solution–diffusion and advection regimes.	Shows an approximately monotonic increase in water flux and overall solvent permeation rates.	Significantly enhances anion selectivity Cl^−^/SO_4_^2−^ SF increases from 4.0 to 23.4), whereas cation selectivity (Na^+^/Mg^2+^) remains stagnant (~1.1 to 1.2).	Custom Piperazine-based (PIP) Polyamide	[15]
Current Density	Operation beyond the overlimiting current density threshold in ED triggers extreme concentration polarization, localized Joule heating, and interfacial water splitting.	Shifts the transport current toward water-splitting products (H^+^ and OH^−^) rather than target species.	Severe collapse of monovalent cation selectivity (e.g., K^+^/Mg^2+^ SF plummets from >1000 at 1.27 mA/cm^2^ to just 22 at elevated currents).	Polyelectrolyte multilayer-coated (PSS/PAH) Nafion^®^	[19]
Operating pH (Polyamides)	Extremes of pH (3 and 12) alter the protonation states of carboxylic (-COOH) and amine (-NH_2_) groups, inducing strong intra-chain electrostatic repulsion and matrix swelling.	Expands the effective pore radius by up to 60%, yielding a 10% to 30% increase in water permeability compared to neutral pH 56.	Amplifies volumetric charge density by 4 to 5 times, enhancing divalent ion rejection via Donnan exclusion and offsetting the physical pore expansion.	Custom Piperazine-based (PIP) Polyamide	[15]
Operating pH (Chelating PIMs)	Exposing amidoxime- or carboxyl-functionalized PIMs to strong alkaline conditions (1 M NaOH) deprotonates the chelating groups, weakening internal hydrogen-bonding networks.	Promotes excessive hydration-driven swelling, leading to a loose network structure and elevated water uptake (up to 60 wt%).	May suppress species-specific ion selectivity in electrodialysis due to the loss of restricted sub-nanometer pore gates.	Deprotonated amidoxime-PIM (AO-PIM-1-De)	[33]
Hydration/State of Water	Increasing the fraction of nonfreezable (bound) water relative to freezable water via hydrophilic backbones (esters/hydroxyls) that interact strongly with water molecules.	Selectively suppresses the diffusion coefficients of co-ions in the membrane phase by restricting bulk-like water pathways.	Simultaneously enhances thermodynamic partitioning selectivity (dielectric exclusion) and diffusion selectivity under high salinity (1 to 5 molal NaCl).	Hydrophilic cross-linked IEMs (SPA-GDMA)	[30]
Membrane Thickness	Restricting the selective layer thickness of nonporous, ligand-functionalized polyelectrolytes, where ion transport is governed by coordination chemistry binding energies.	Strongly binds target species (Cu^2+^), exhibiting thickness-dependent flux, whereas weaker-binding species (Co^2+^) display thickness-independent transport.	Exceptionally high selectivities (Cu^2+^ /Co^2+^, SF > 23) are achieved at ultrathin limits, whereas thick commercial-scale membranes drop to SF < 5.	Iminodiacetate-functionalized multilayers (PDCMAA/PAH)	[12]
Exposure Duration (Ionic Liquids)	Prolonged exposure to aggressive acidic media (1 M HCl for up to 85 days/2048 h) leads to physical phase-separation and progressive loss of physically entrapped ionic liquids.	Causes a progressive decline in active transport sites and transition-metal extraction flux.	Compromises long-term selectivity; unstable quaternary ammonium carriers ([MTOA^+^][Cl^−^]) lose over 50% mass, whereas compatible carriers ([omim^+^][PF6^−^]) retain 82%.	Poly(vinyl chloride) (PVC)-based PIMs	[51]
Exposure Duration (Neutral Chelators)	Repeated lithium extraction/back-extraction cycles in acidic solutions cause progressive leaching of neutral carriers into contiguous aqueous phases.	Lithium recovery efficiency drops by 17% in the 3rd cycle and over 60% after 5 cycles (or 40% reduction for TBP/FeCl_3_ carriers).	Rapid decay of monovalent/divalent separation factors across successive operating runs due to carrier depletion.	Cellulose triacetate (CTA)/PVC-based PIMs	[24]

Note: The reported trends are compiled from studies with heterogeneous membrane chemistries, operating conditions, and performance definitions; therefore, values should be interpreted as directionally comparable rather than directly comparable unless the same driving force, feed composition, and metric definition are explicitly reported.

## Data Availability

No data were generated or analyzed in this study. This article is a review based exclusively on the previously published literature, which is cited throughout the manuscript.

## Nomenclature

2D	Two-dimensional
AEM	Anion exchange membrane
AI	Artificial Intelligence
AIMD	Ab initio molecular dynamics
CAIP-MOF	Charge-asymmetry-induced permselectivity metal–organic framework
CEM	Cation exchange membrane
CFD	Computational fluid dynamics
CMM	Charge-mosaic membrane
COF	Covalent organic framework
CVD	Chemical vapor deposition
D2EHPA	Di-(2-ethylhexyl) phosphoric acid
DB18C6	Dibenzo-18-crown-6
DFT	Density Functional Theory
DMBP-QTB	Dimethylbiphenyl quaternized Tröger’s base
DMF	Dimethylformamide
DSPM-DE	Donnan steric pore model with dielectric exclusion
ED	Electrodialysis
EDTA	Ethylenediaminetetraacetic acid
FAIR	Findable, Accessible, Interoperable, and Reusable
FCS	Fluorescence correlation spectroscopy
GNN	Graph neural network
GO	Graphene oxide
HMO	Hydrogenated manganese oxide
IDA	Iminodiacetic acid
IEM	Ion-exchange membrane
IL	Ionic liquid
LbL	Layer-by-layer
LCA	Life cycle analysis
LLM	Large language model
LSRRO	Low-salt-rejection reverse osmosis
ML	Machine Learning
MOF	Metal–organic framework
MTOACl/[MTOA^+^][Cl^−^]	Methyltrioctylammonium chloride
MWCO	Molecular weight cut-off
NF	Nanofiltration
NMP	N-Methyl-2-pyrrolidone
OMD	Open membrane database
[omim^+^][PF6^−^]	1-Octyl-3-methylimidazolium hexafluorophosphate
PA-ZNs	Polyamide zwitterionic nanochannels
PEI	Polyethylenimine
PFOAM50	Pore-filled trioctylamine membrane (50% functionalization)
PIM	Polymer inclusion membrane
PILIM	Polymer–ionic liquid inclusion membrane
PINN	Physics-Informed Neural Network
PLA	Polylactic acid
PRISMA	Preferred reporting items for systematic reviews and meta-analyses
PS-DVB	Polystyrene-divinylbenzene
PSS	Poly(sodium 4-styrenesulfonate)
PVC	Polyvinyl chloride
PVDF	Polyvinylidene fluoride
PVDF−HFP	Poly(vinylidene fluoride-co-hexafluoropropylene)
QPInM15	Quaternized Polymer of Intrinsic Microporosity (15% functionalization)
RO	Reverse osmosis
SF	Separation factor
SHAP	SHapley additive exPlanations
SPEEK	Sulfonated poly(ether ether ketone)
SPES	Sulfonated polyethersulfone
TB	Tröger’s Base
TDS	Total Dissolved Solids
TEA	Techno-economic assessment
TFC	Thin-film composite
TPA100	Terephthalaldehyde-crosslinked Tröger’s base membrane (100% crosslinking)
TST	Transition State Theory
XAI	Explainable artificial intelligence
XGBoost	Extreme Gradient Boosting
ZCDM	Zwitterionic cyclodextrin membrane
ZIF-8	Zeolitic imidazolate framework-8
ZIOS	Zinc iron oxide sulfide
